# COVID-19 in the Initiation and Progression of Atherosclerosis: Pathophysiology During and Beyond the Acute Phase

**DOI:** 10.1016/j.jacadv.2024.101107

**Published:** 2024-07-17

**Authors:** Vignesh Chidambaram, Amudha Kumar, Murrium I. Sadaf, Emily Lu, Subhi J. Al’Aref, Tushar Tarun, Panagis Galiatsatos, Martha Gulati, Roger S. Blumenthal, Thorsten M. Leucker, Petros C. Karakousis, Jawahar L. Mehta

**Affiliations:** a Department of Internal Medicine, University of Arkansas for Medical Sciences, Little Rock, Arkansas, USA; b Division of Cardiology, Department of Medicine, Loyola University Medical Center, Maywood, Illinois, USA; c Division of Cardiovascular Medicine, Department of Medicine, University of Arkansas for Medical Sciences, Little Rock, Arkansas, USA; d Division of Infectious Diseases, Department of Medicine, Johns Hopkins School of Medicine, Baltimore, Maryland, USA; e Division of Pulmonary and Critical Care Medicine, Department of Medicine, Johns Hopkins School of Medicine, Baltimore, Maryland, USA; f Barbra Streisand Women's Heart Center, Smidt Heart Institute, Cedars-Sinai Medical Center, Los Angeles, California, USA; g Ciccarone Center for the Prevention of Cardiovascular Disease, Johns Hopkins University School of Medicine, Baltimore, Maryland, USA; h Department of International Health, Johns Hopkins Bloomberg School of Public Health, Baltimore, Maryland, USA; i Department of Molecular Microbiology and Immunology, Johns Hopkins Bloomberg School of Public Health, Baltimore, Maryland, USA; j Division of Cardiovascular Medicine, Central Arkansas Veterans Healthcare System, Little Rock, Arkansas, USA

**Keywords:** atherosclerotic cardiovascular disease, endothelial dysfunction, inflammation, platelet activation, SARS-CoV-2

## Abstract

The incidence of atherosclerotic cardiovascular disease is increasing globally, especially in low- and middle-income countries, despite significant efforts to reduce traditional risk factors. Premature subclinical atherosclerosis has been documented in association with several viral infections. The magnitude of the recent COVID-19 pandemic has highlighted the need to understand the association between SARS-CoV-2 and atherosclerosis. This review examines various pathophysiological mechanisms, including endothelial dysfunction, platelet activation, and inflammatory and immune hyperactivation triggered by SARS-CoV-2 infection, with specific attention on their roles in initiating and promoting the progression of atherosclerotic lesions. Additionally, it addresses the various pathogenic mechanisms by which COVID-19 in the post-acute phase may contribute to the development of vascular disease. Understanding the overlap of these syndromes may enable novel therapeutic strategies. We further explore the need for guidelines for closer follow-up for the often-overlooked evidence of atherosclerotic cardiovascular disease among patients with recent COVID-19, particularly those with cardiometabolic risk factors.

## Highlights

• SARS-CoV-2 infection can markedly influence the initiation and progression of atherosclerotic lesions.
• Endothelial dysfunction, platelet activation, and persistent inflammation are potential drivers of increased atherosclerosis following COVID-19.
• Understanding the pathogenesis of atherosclerosis in COVID-19 can provide insights into cardiovascular disease mechanisms in other chronic infections.
• Recognizing the cardiovascular implications of long COVID-19 highlights the importance of proactive risk management and advocates for further research into this topic.

Atherosclerosis, with its major long-term complications such as myocardial infarction (MI) and stroke, is increasing globally, especially in low- and middle-income countries.^1^ Despite efforts targeting traditional risk factors, the incidence of atherosclerotic cardiovascular disease (ASCVD)^2^ remains high. Notably, even young individuals and those without classical risk factors may develop ASCVD.^3^

Premature subclinical atherosclerosis is documented in patients with HIV, hepatitis C, cytomegalovirus, influenza, and tuberculosis.^4^^,^^5^ Beyond direct pathogen effects, the resultant systemic or organ-specific inflammatory response may drive atherosclerosis initiation and progression.^6^ Furthermore, inflammation can trigger a local vascular reaction within arterial plaques, resulting in plaque disruption, thrombosis, and acute ischemic events.^6^

Considering the global impact of the COVID-19 pandemic,^7^ it is imperative to understand its association with atherosclerosis. Endothelial cell (EC) dysfunction, an early event, and the subsequent platelet activation and adhesion to the activated endothelium are central to both atherosclerosis^3^ and COVID-19,^8^^,^^9^ suggesting a major link between them. Though other viral infections^10^ can trigger pro-inflammatory cytokine release, the immune overactivation in SARS-CoV-2 infection is particularly severe, enhancing EC dysfunction and perpetuating this vicious cycle.^8^

Our review focuses on the various pathophysiological mechanisms triggered by SARS-CoV-2 infection that can initiate and promote atherosclerotic lesions. Given the high frequency of persistent symptoms and sequelae in patients who recovered from COVID-19,^11^ we also explore the pathogenesis of vascular disease in long-COVID-19 and the need for guidelines for follow-up and evaluation of ASCVD, particularly among those with cardiometabolic risk factors.

### Data sources and search strategy

To understand the interconnected mechanisms between COVID-19 and atherosclerosis, we conducted a comprehensive search of literature published up to July 2023 in PubMed and Embase and updated it in February 2024. We used Medical Subject Headings and keywords in various combinations, including “COVID-19,” “SARS-CoV-2,” and/or “atherosclerosis,” “arteriosclerosis,” “coronary artery disease,” “vascular,” “ASCVD,” “coronary syndrome,” “myocardial infarction,” “ischemia,” and/or “pathophysiology,” “mechanisms,” “etiology.” For exploring mechanisms specific to long-COVID-19, we used “long-COVID-19,” “long-haul COVID,” “post-acute COVID,” “persistent COVID-19,” “post-acute sequelae of SARS-CoV-2,” “chronic COVID,” and “COVID-19” in conjunction with “follow-up,” “postinfection,” or “sequelae.” Additionally, we reviewed reference lists from pertinent reviews and editorials to ensure a thorough exploration of the topic.

## Clinical coronary syndromes during the acute phase

Patients with pre-existing cardiovascular disease and risk factors have a worse prognosis with COVID-19.^12^^,^^13^ Conversely, the acute phase of COVID-19 is linked to acute ischemic events.^14^ Around 20% of hospitalized patients with COVID-19 exhibit myocardial injury, as evidenced by elevated cardiac troponins,^15^ likely secondary to plaque rupture, coronary spasm, microthrombi, myocarditis, cytokine storm, or direct endothelial or vascular injury. Although individual autopsy studies reveal varying observations regarding lymphocytic myocarditis in COVID-19-associated myocardial injury,^16^^,^^17^ a systematic review of cardiac findings from postmortem studies identified myocardial cell necrosis and myocardial edema as the most common findings, with instances of focal or multifocal myocarditis being relatively minor.^18^ The systematic review also notes a median prevalence of 36.2% for microthrombi and 11.8% for acute MI.^18^ In 2 case series, nearly 30 to 40% COVID-19 patients with ST-segment elevation myocardial infarction (STEMI) showed nonobstructed coronaries on invasive angiography.^14^^,^^19^ A North American registry with 1,185 patients^20^ highlighted the absence of a culprit artery in almost 20% of patients undergoing angiography for STEMI with confirmed or suspected COVID-19.^20^ The findings suggest viral effects beyond plaque destabilization, highlighting direct and indirect pathways to cardiac injury.

STEMI patients with concurrent COVID-19 faced higher risks, including in-hospital death, stroke, recurrent MI, or repeat unplanned revascularization compared to matched pre-COVID-19 STEMI patients.^20^ A UK retrospective cohort of patients with STEMI reported that those with concurrent COVID-19 had higher troponin levels, modified thrombus grades, and rates of multivessel thrombosis, as well as increased use of glycoprotein IIb/IIIa inhibitors than those without COVID-19.^21^ Atherosclerotic plaque rupture and arterial thrombus formation in these patients are likely due to EC dysfunction, platelet activation, and concomitant systemic inflammation.^22^ These very similar mechanisms are also key to the initiation and progression of atherosclerosis and are further explored below.

## Endothelial dysfunction

### Role in atherosclerosis

**Figure 1:**
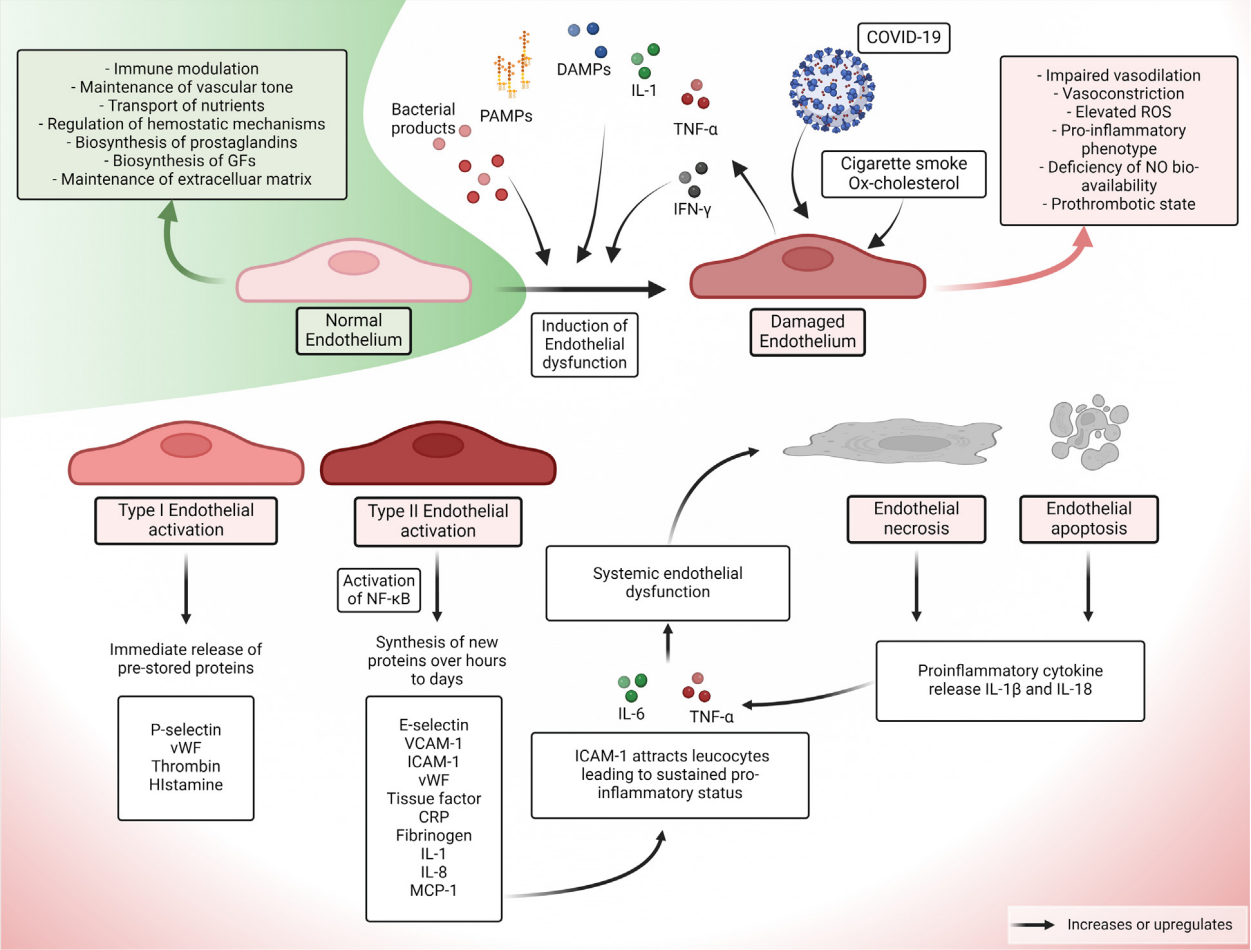
**Endothelial Dysfunction in COVID-19** PAMPs, DAMPs, and pro-inflammatory cytokines, including IL-1, TNF-α, and IFN-γ, may induce either reversible endothelial activation (type I and II) or cell injury (apoptosis and necrosis). Created using Biorender.com. CRP = C-reactive protein; DAMP = damage-associated molecular pattern; ICAM = intercellular adhesion molecule; IFN = interferon; IL = interleukin; MCP = monocyte chemoattractant protein; NF-κB = nuclear factor-κB; Ox-cholesterol = oxidized cholesterol; PAMP = pathogen-associated molecular pattern; TF = tissue factor; TNF = tumor necrosis factor; VCAM = vascular cell adhesion molecule; vWF = von-Willebrand factor.

The endothelium has important functions in inflammation, immune modulation, vascular tone maintenance, and hemostasis.^23^ EC dysfunction, an early step in atherosclerosis preceding clinical symptoms, can be triggered by oxidized cholesterol, hyperglycemia, infection, inflammation, and hemodynamic processes.^24^ Notably, pathogen- and damage-associated molecular patterns (PAMPs and DAMPs, respectively), pro-inflammatory cytokines (eg, interleukin [IL]-1, tumor necrosis factor [TNF]-α, and interferon [IFN]-γ) can induce either reversible EC activation (type I and II) or EC injury (apoptosis and necrosis)^24^ (Figure 1). In type I EC activation (self-limited), immediate release of prestored proteins occurs,^25^ while type II (modulation of the functional phenotype) results in activation of transcriptional factors like nuclear factor-κB (NF-κB),^25^ and de-novo synthesis of E-selectin, P-selectin (CD62P), intercellular adhesion molecule (ICAM)-1 or CD54, vascular cell adhesion molecule (VCAM)-1 or CD106, von-Willebrand factor, tissue factor (TF), IL-1, IL-8, monocyte chemoattractant protein (MCP)-1, and fibrinogen. The endothelium subsequently may undergo cell death (apoptosis or necrosis), increasing inflammatory mediators and further exacerbating endothelial damage.^25^

Vascular inflammation ensues when activated EC express CAMs and secrete cytokines (TNF-α, IL-6), and smooth muscle cells (SMCs) secrete chemokines and chemoattractants (MCP-1/CCL2).^25^ This promotes platelet activation and attracts monocytes, lymphocytes, and neutrophils, facilitating subsequent transmigration and focal recruitment to the subendothelial space.^26^ Monocytes later differentiate into macrophages, forming foam cells on oxidized lipoprotein uptake.^26^ These mechanisms induce SMC proliferation and extracellular matrix synthesis within the intima, resulting in the classical fibromuscular atherosclerotic plaque. The above mechanisms underscore the importance of EC dysfunction in the pathogenesis of atherosclerosis.

### EC dysfunction in COVID-19

EC dysfunction is central in the pathogenesis of COVID-19, primarily affecting blood vessels in the heart, lungs, and brain.^8^ EC dysfunction triggers the release of inflammatory mediators and upregulates adhesion molecules on ECs, leading to leukocyte adherence and transmigration that compromise vascular barrier integrity, contributing significantly to the acute phase of COVID-19.^8^ These changes can precipitate macrovascular and microvascular events that impair organ perfusion and exacerbate the severity of COVID-19. SARS-CoV-2 is proposed to damage the endothelium either via direct infection of ECs^27^^,^^28^ or via the more accepted indirect interaction with EC^29^ through circulating mediators and immune mechanisms.

### Direct interaction of SARS-CoV-2 with EC

Among COVID-19 patients with evidence of endothelitis, Varga et al observed virus-like particles in glomerular ECs with surrounding inflammatory cells.^30^ In contrast, others found EC injury without discernible virions in cells using electron microscopy.^31^^,^^32^ While the presence of EC dysfunction in COVID-19 is widely accepted, the role of direct endothelial infection in COVID-19 pathogenesis remains debated, with inconsistent findings supporting both angiotensin-converting enzyme 2 (ACE2)-dependent and independent mechanisms.

#### Role of ACE2

ACE2 converts angiotensin (Ang) II to Ang 1 to 7; this induces vasodilation, reduces vascular inflammation^33^ and endothelial reactive oxygen species (ROS),^34^ and enhances plaque stability through decreased matrix metalloprotease-2 and matrix metalloprotease-9.^33^ Furthermore, ACE2 inhibits NF-kB signaling, decreasing the secretion of pro-inflammatory cytokines (TNF-α and IL-6), and attenuating leukocyte adhesion through reduced expression of VCAM-1 and MCP-1.^34^

**Figure 2:**
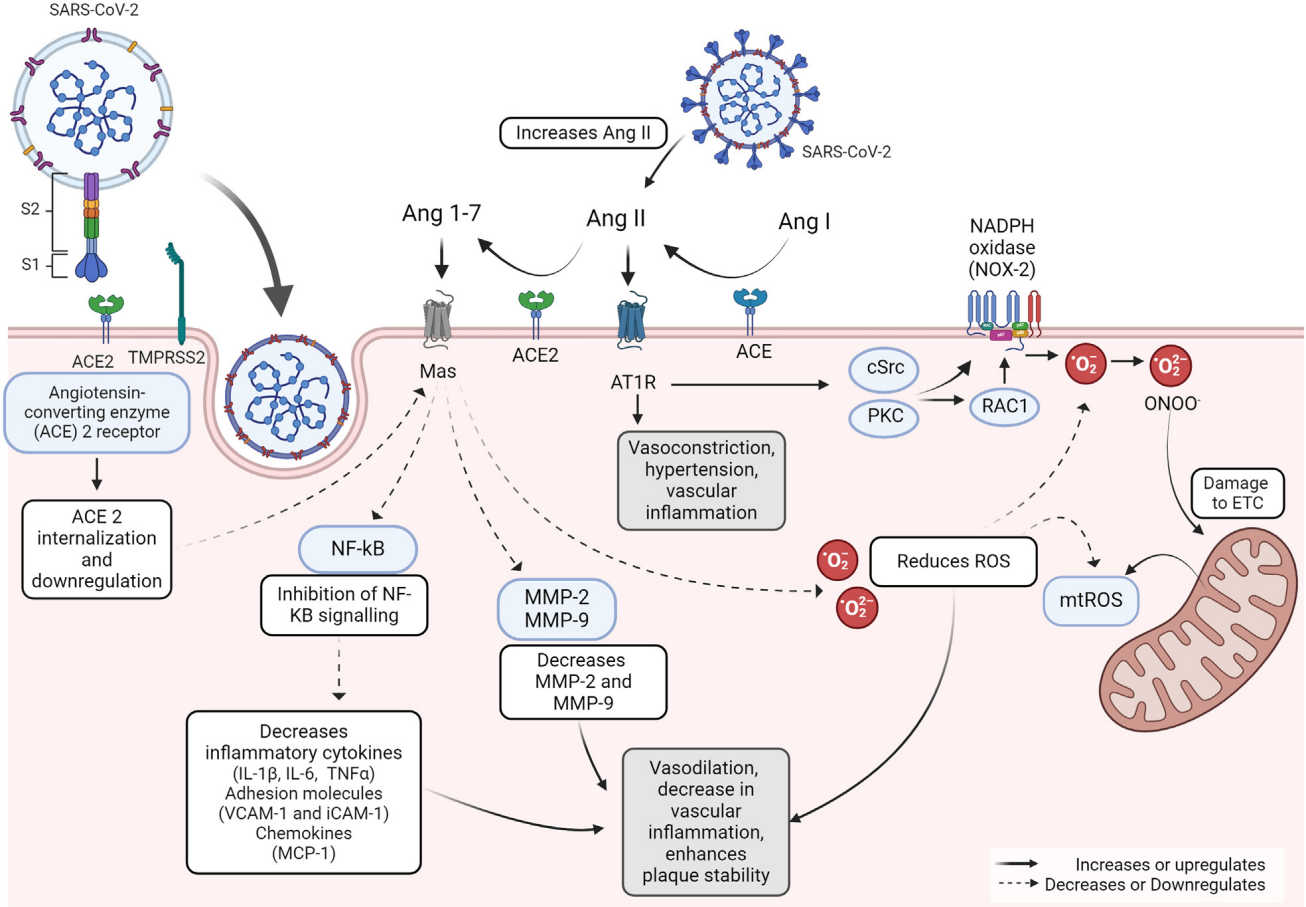
**Role of ACE2 and Oxidative Stress in COVID-19** Ang II Is converted by ACE2 to Ang 1 to 7, leading to vasodilation, reduced vascular inflammation, and enhanced plaque stability through decreased endothelial production of ROS, MMP-2, and MMP-9, and inhibition of NF-kB signaling. SARS-CoV-2 binding to ACE2 and subsequent cellular entry results in ACE2 internalization and loss of its catalytic activity in the renin-Ang system, increasing Ang II levels and promoting an inflammatory, Procoagulant, and apoptotic endothelial phenotype. Major ROS-producing systems in the vascular wall include NOX-2, xanthine oxidase, the mitochondrial respiratory chain, and uncoupled eNOS. SARS-CoV-2 infection, via increased Ang-II, activates the AT1R receptor, leading to PKC and c-SRC activation, stimulating NOX-2 and increasing superoxide anion production and mitochondrial ROS release, thereby reducing NO bioavailability. Activated endothelial superoxide anions contribute to local oxidative stress, facilitating key atherogenesis events. Created Using Biorender.com. ACE = angiotensin-converting enzyme; Ang = angiotensin; AT1R = angiotensin II receptor type 1; c-Src = cytoplasmic-Rho family GTPase; eNOS = endothelial nitric oxide synthase; IL = interleukin; MCP = monocyte chemoattractant protein; MMP = matrix metalloprotease; mtROS = mitochondrial reactive oxygen species; NF-κB = nuclear factor-κB; NO = nitric oxide; NOX-2 = nicotinamide adenine dinucleotide phosphate oxidase-2; PKC = protein kinase C; ROS = reactive oxygen species; TNF = tumor necrosis factor; VCAM = vascular cell adhesion molecule.

In various cells, SARS-CoV-2 binding to membrane-bound ACE2 may result in ACE2 internalization, conferring an inflammatory phenotype by disrupting ACE2 activity in the renin-Ang system and increasing Ang II (Figure 2).^28^ Concurrently, SARS-CoV-2 promotes ACE2 ectodomain shedding, generating soluble ACE2,^35^ which is more abundant in the circulation of patients with vascular disease.^36^ Though various human tissues express ACE2 mRNA, its expression in vascular ECs is debated.^37^^,^^38^

Early studies showed that SARS-CoV-2 infects ECs via surface ACE2,^27^^,^^28^ which is inhibited by recombinant human ACE2.^27^ When SARS-CoV-2 was inoculated in vitro into ECs from various human tissues, SARS-CoV-2 spike (S) protein was uniquely detected in coronary artery ECs, which was attributed to their ACE2 expression.^39^ Furthermore, high-density lipoprotein-scavenger receptor B type 1,^40^ expressed in various cell types, including ECs, facilitates ACE2-mediated SARS-CoV-2 entry.

However, other clinical, transcriptomic, and epigenetic data question whether the virus directly infects ECs via ACE2,^38^^,^^41^ citing very low or absent basal or inducible ACE2 expression in ECs compared to respiratory or gastrointestinal cells.^38^ Occasional ACE2 expression (usually a single transcript) in ECs might reflect true expression or contamination from adherent pericyte fragments.^38^ Further high-resolution microscopy studies are needed to determine the cellular source of ACE2 in the vasculature. Despite early hypotheses linking ACE inhibitors to increased SARS-CoV-2 infection risk by increasing endothelial ACE2 expression, current evidence refutes increased COVID-19 risk or severity among patients taking these drugs.^42^ Furthermore, it is essential to note that future studies are needed to determine whether modulation of ACE2-SARS-CoV-2 interaction or the renin-angiotensin system could mitigate ASCVD following COVID-19.

#### ACE2-independent cell surface receptors

Several ACE2-independent cell surface receptors have been proposed for SARS-CoV-2. Neuropilins, highly expressed in ECs and epithelial cells, influence vascular permeability and immune regulation.^43^ Neuropilin 1, a coreceptor of vascular endothelial growth factor in ECs, is implicated in SARS-CoV-2 infectivity via interactions with unique Furin-generated substrates of S1.^43^ CD147, another EC receptor, facilitates dose-dependent SARS-CoV-2 entry in ACE2-deficient cells.^44^

While these EC surface receptors may represent alternate viral entry pathways, further in-vivo validation is needed. As detailed below, current evidence points more toward indirect mechanisms, including immune cell and platelet activation, as well as increased circulating pro-inflammatory cytokines, for the EC dysfunction observed in severe COVID-19 patients.^45^

### Signaling pathways and other mechanisms in COVID-19-associated EC dysfunction

#### Activation of NF-κB

The transcription factor NF-κB, crucial in EC activation toward a pro-inflammatory phenotype, is primed for greater activation in atherosclerosis-prone arterial regions.^46^ Several pathogenic stimuli for EC dysfunction stimulate pattern recognition receptors (PRRs) like Toll-like receptors (TLRs)^46^ and depend on subsequent NF-κB signaling. This induces the expression of pro-inflammatory cytokines (IL-1, IL-6, IL-8, IL-12, and TNF-α), adhesion molecules, and chemokines (MCP-1, IL-18, and RANTES), driving atherosclerosis via EC dysfunction, leukocyte infiltration, and SMC migration and proliferation.^47^

While mechanisms of NF-κB activation, such as increased oxidative stress seen in other viral infections, including influenza,^48^ HIV,^49^ and HTLV-1,^50^ are also present in COVID-19,^51^ pathways unique to SARS-CoV-2 infection exist. The SARS-CoV-2 S protein promotes NF-κB nuclear translocation through IκBα degradation, increasing expression of adhesion molecules, FVIII, TF, and pro-inflammatory cytokines (TNF-α, IL-1β, and IL-6).^52^ Furthermore, the SARS-CoV-2 nucleocapsid (N) protein enhances the association between the TAK1 (transforming growth factor-β activated kinase 1) and IKK (IκB kinase) complex, facilitating NF-κB hyperactivation.^53^ On the other hand, ACE2-deficient ECs, resistant to SARS-CoV-2 infection, showed increased activation compared to those expressing ACE2, possibly due to NF-kB upregulation following TLR4 activation; conversely, a TLR4 antagonist inhibited this activation.^54^ Furthermore, the SARS-CoV-2 S and N proteins activate ECs through TLR-2/NF-κB and mitogen-activated protein kinase pathways, inducing inflammation without viral entry.^55^

Such NF-κB upregulation in COVID-19, through the aforementioned pathways, subsequently potentiates the inflammatory response.^52^ Furthermore, NF-κB modulates the NLRP3 (nucleotide-binding domain, leucine-rich-containing family, pyrin domain-containing-3) inflammasome, enhancing immune activation in COVID-19.^56^ Although NF-κB activation may play a role in the pathogenesis of COVID-19-related atherosclerosis, future studies are needed to evaluate whether drugs modulating NF-κB are beneficial in this aspect.

#### Increased expression of VCAM-1 and ICAM-1

The expression of VCAM-1 and ICAM-1, which are localized to the endothelium in atherosclerosis-susceptible arterial regions, precedes monocyte recruitment and forms important links between inflammation and atherosclerosis.^57^^,^^58^ Monocytes and lymphocytes bind to VCAM-1 on ECs via the counter-receptor VLA-4 (integrin α4β1) and to ICAM-1 via LFA-1(integrin αLβ2).^57^^,^^58^ Plasma levels of soluble sVCAM-1 and sICAM-1 correlate with atherosclerotic lesion burden^59^ and predict future cardiovascular events.^59^

In COVID-19, elevated plasma levels of sVCAM-1 and sICAM-1 correlate with disease severity.^60^ While the precise mechanism of elevation of ICAM-1 and VCAM-1 in SARS-CoV-2 infection is unclear, it appears to be driven by virus-induced inflammatory response and EC activation.^61^ Severe COVID-19 triggers a cytokine storm, leading to upregulation of these adhesion molecules, facilitating leukocyte adhesion and migration, exacerbating vascular inflammation. Rotoli et al^62^ showed that SARS-CoV-2 spike protein activates EC, with pro-inflammatory mediators from spike-activated macrophages further amplifying this activation, thereby leading to upregulation of VCAM-1 and ICAM-1. Elevated VCAM-1 levels persisted in COVID-19 patients for several months, normalizing after a year,^63^ while ICAM-1 levels remained elevated 16 months postinfection,^64^ indicating persistent EC activation. The effect of ICAM inhibitors, such as Resveratrol, on COVID-19-associated atherosclerosis is yet to be explored.

#### Nitric oxide and oxidative stress

Major vascular ROS–producing systems include nicotinamide adenine dinucleotide phosphate oxidase-2, the mitochondrial respiratory chain, xanthine oxidase, and uncoupled endothelial nitric oxide (NO) synthase.^65^ Oxidative stress, augmented by cardiometabolic risk factors, is countered by superoxide dismutase and glutathione.^66^ EC dysfunction enhances oxidative stress, facilitating key atherosclerosis events, like oxidative modification of phospholipids and lipoproteins and macrophage activation, while endothelial NO inhibits them.^65^^,^^66^

SARS-CoV-2 increases superoxide anion production and mitochondrial DNA release, leading to TLR9 and NF-κB activation,^67^ increased oxidative stress, and decreased NO bioavailability.^68^ In ECs, AT1R activation stimulates ROS generation via nicotinamide adenine dinucleotide phosphate oxidase-2, limiting NO bioavailability.^69^ While endothelial NO synthase constitutively produces endothelial NO, inducible NO synthase activation induces higher levels during inflammation. Montiel et al observed elevated NO levels in a pre-COVID-19 septic shock cohort compared to those with COVID-19, possibly due to neutrophil activation and cytokine-dependent inducible NO synthase^68^ induction in sepsis, while suggesting unique oxidative stress mechanisms unrelated to renin-Ang system hyperactivity or neutrophil activation in COVID-19.^68^

## Platelet activation

### Role of platelets in atherosclerosis

Platelets and platelet-derived factors play well-characterized roles in atherosclerosis initiation and progression^70^: 1) platelet adhesion to endothelium and formation of platelet-leukocyte heterotypic aggregates, enabling leukocyte transmigration; and 2) release of pro-inflammatory cytokines and chemokines, such as platelet factor 4 (PF4), by activated platelets and platelet-monocyte aggregates, activating ECs and innate immune cells.^71^

### Platelets and COVID-19

Though the majority of platelets becomes hyperactivated in COVID-19,^9^^,^^72^^,^^73^ a small fraction becomes functionally defective.^74^ The mechanisms of interaction between SARS-CoV-2 and platelets and/or megakaryocytes remain a subject of debate.

#### Direct interaction and internalization of SARS-CoV-2 into platelets

Direct interaction of SARS-CoV-2 with platelets and hyperactivation was proposed following SARS-CoV-2 RNA detection in platelets of COVID-19 patients.^9^^,^^72^ Although an ACE2-dependent^75^ interaction was hypothesized, platelet ACE2 expression remains unconfirmed.^9^^,^^72^ Other studies indicated ACE2-independent mechanisms,^76^ including: 1) direct interaction with other platelet receptors, such as CD147,^44^ Fc receptor for IgG (FcγRIIA), CD26,^9^ CD42b,^77^ and TLR7 in endocytosed vesicles; and 2) nonreceptor pathways, like micropinocytosis and phagocytosis of SARS-CoV-2-containing apoptotic cell-fragments or microparticles into platelets.^78^

Koupenova et al^79^ demonstrated the ability of platelets to internalize SARS-CoV-2 without supporting replication, leading to viral degradation. These platelets fail to activate and undergo morphological changes, like membrane budding and ultimately programmed cell death,^79^ partly explaining the thrombocytopenia in COVID-19 patients. Released extracellular vesicles from these platelets further amplify immune and cytokine dysregulation.^74^

#### Indirect interactions with platelets

Despite divergent mechanisms proposed for SARS-CoV-2 platelet interactions, consensus exists that platelet activation occurs during COVID-19. Puhm et al observed no platelet activation even at high viral concentrations without coagulation factors, suggesting that direct platelet–SARS-CoV-2 interactions might be too rare to explain platelet hyperactivation.^80^ Instead, in COVID-19 patients, elevated TF levels, arising from activated ECs and macrophages,^81^ initiate the coagulation cascade with subsequent thrombin generation, which, even under low concentrations, may potentially activate platelets via protease-activated receptor-1 and -4.^82^

#### Platelet adhesion and activation, CD40L, and P-selectin

**Figure 3:**
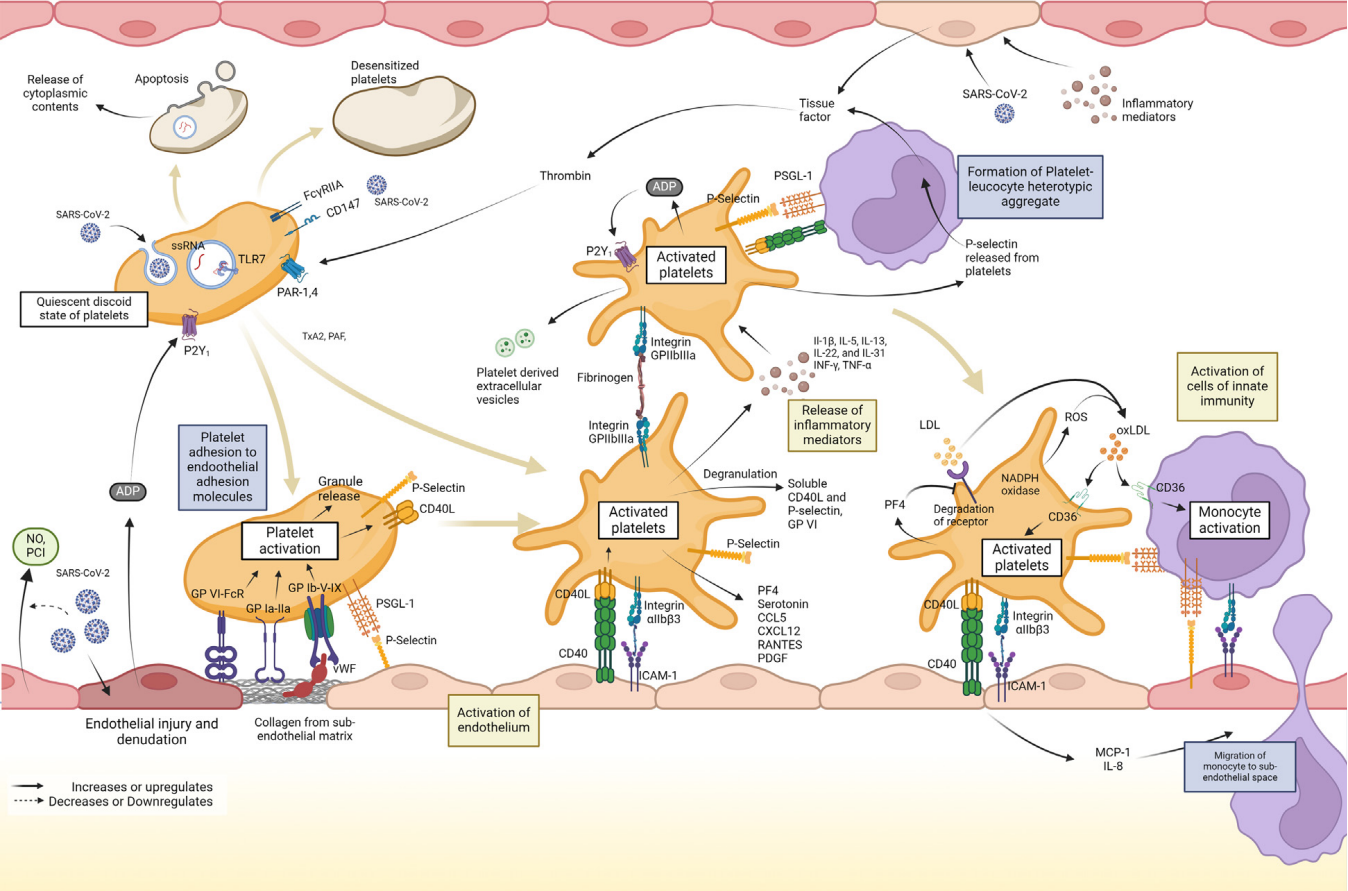
**Platelet Activation in COVID-19** Key mechanisms for platelet activation in atherosclerosis include: 1) platelet adhesion to endothelium via cell-adhesion molecules, forming platelet-leukocyte aggregates that promote monocyte migration into the intima, and 2) release of inflammatory mediators by activated platelets that stimulate the vascular endothelium and innate immune cells. SARS-CoV-2-platelet interaction occurs through ACE2-dependent and independent mechanisms, such as other platelet receptors or non-receptor pathways, initiating platelet adhesion and activation. Activated platelets undergo a conformational change to a stellate appearance, facilitating leukocyte recruitment. Platelet activation leads to degranulation, and release of alpha and dense granules, and platelet extracellular vesicles (EVs). SARS-CoV-2 infection also triggers platelet release of pro-inflammatory cytokines, modulating the local immune response. Created Using Biorender.com. ADP = adenosine diphosphate; CCL = C-C motif ligand; CRP, C-reactive protein; CXCL = C-X-C motif ligand; FcR = Fc receptor gamma chain; GP = glycoprotein; Fc receptor for IgG (FcγRIIA); ICAM = intercellular adhesion molecule; IFN = interferon; IL = interleukin; MCP = monocyte chemoattractant protein; NO = nitric oxide; PAR = protease-activated receptor; PCI = prostacyclin; PDGF = platelet-derived growth factor; PF = platelet factor; PSGL = P-selectin glycoprotein ligand; RANTES = Regulated Upon Activation, Normal T Cell Expressed and Presumably Secreted Chemokine; ssRNA = single-stranded RNA; TF = tissue factor; TLR = Toll-like receptor; TNF = tumor necrosis factor; VCAM = vascular cell adhesion molecule; vWF = von-Willebrand factor.

EC dysfunction leads to NO-prostacyclin imbalance, as well as upregulation of endothelial CAMs, promoting platelet adhesion.^25^ Following adhesion at the site of EC activation (to P-selectin, ICAM-1, etc), or EC injury (to subendothelial collagen via platelet glycoprotein VI),^25^ platelets assume the classical activated stellate appearance (Figure 3). Activated platelets express markers like P-selectin, CD40L, TLRs, FcγRIIA, and activated integrin GP IIb IIIa, enhancing aggregation, degranulation (serotonin and PF4), and the release of extracellular vesicles.^72^ CD40 L on activated platelets triggers EC activation and secretion of MCP-1 and IL-8.^83^ MCP-1 production, additionally attributed to endothelial NF-ĸB activation by platelet-derived IL-1β^84^ and platelet adhesion to SMCs, augments SMC migration, which is crucial for atherogenesis.

Interactions between platelet P-selectin and leukocyte P-selectin glycoprotein ligand-1 facilitate leukocyte transmigration^85^ and influence plaque initiation and their cellularity. Interaction with P-selectin glycoprotein ligand-1 stimulates P-selectin shedding,^85^ with elevated soluble P-selectin (sP-selectin) levels in plasma being linked to an increased risk of MI and stroke.^86^

Regardless of COVID-19 severity, platelets exhibit increased expression of P-selectin and CD40 L,^9^ enhancing platelet-leukocyte interactions and TF expression.^9^^,^^72^^,^^73^ Treatment with crizanlizumab, targeting P-selectin, resulted in sustained P-selectin inhibition and antithrombotic effects in patients with COVID-19.^87^

#### Platelet degranulation and cytokine release

In COVID-19 patients, platelet degranulation leads to the release of sP-selectin, sCD40 L (soluble), PF4, RANTES, serotonin, and sGPVI, correlating with disease severity.^72^^,^^76^ PF4 and RANTES stimulate monocyte differentiation into macrophages,^88^ while serotonin causes vasoconstriction and activation of ECs and platelets (along with ADP).^89^ SARS-CoV-2 induces pro-inflammatory cytokine release from activated platelets, impacting local immune responses.^72^ This might result from altered transcriptional profiles of megakaryocytes, with subsequent transfer of mRNA transcripts to newly formed platelets.^9^ Hence, platelets and ECs mutually amplify the inflammatory response in COVID-19.

Though the role of platelet hyperactivation in thrombosis and coagulopathy in COVID-19 is well established, further research into the above-described putative pathways linking platelet dysfunction in COVID-19 to atherosclerosis is essential to elucidate the precise mechanisms. Additional studies are necessary to determine whether drugs targeting these pathways offer therapeutic benefits in this aspect.

## Role of inflammation and immunity

### Immune response and atherosclerosis

Inflammatory processes, along with the innate and adaptive immune system, drive the initiation and progression of atherosclerosis^3^ and are associated with future cardiovascular events beyond traditional cardiovascular risk factors. Inflammation triggers EC dysfunction, platelet activation, monocyte transmigration, and foam cell formation.^90^ The peripheries of atherosclerotic plaques contain abundant innate (activated macrophages, dendritic cells, and NK-T-Cells) and adaptive (T cells) immune cells. TNF-α and IL-1 are especially relevant, as they promote the expression of other cytokines, CAMs, and vascular SMC migration and mitogenesis.^3^

Although T cells generally exacerbate atherosclerosis (specifically the pro-atherogenic Th1 response), certain subsets limit inflammation and plaque complications. Antigen exposure and IL-12 and IL-18 from macrophages induce Th1 differentiation^91^^,^^92^ and IFN-γ secretion, promoting atherosclerosis via EC junction disruption, foam-cell formation, matrix degradation, and plaque destabilization.^93^ The role of CD8+ T cells in atherosclerosis remains unclear, while Treg cells have well-known anti-atherosclerotic properties.^94^

### Innate immunity and COVID-19

**Figure 4:**
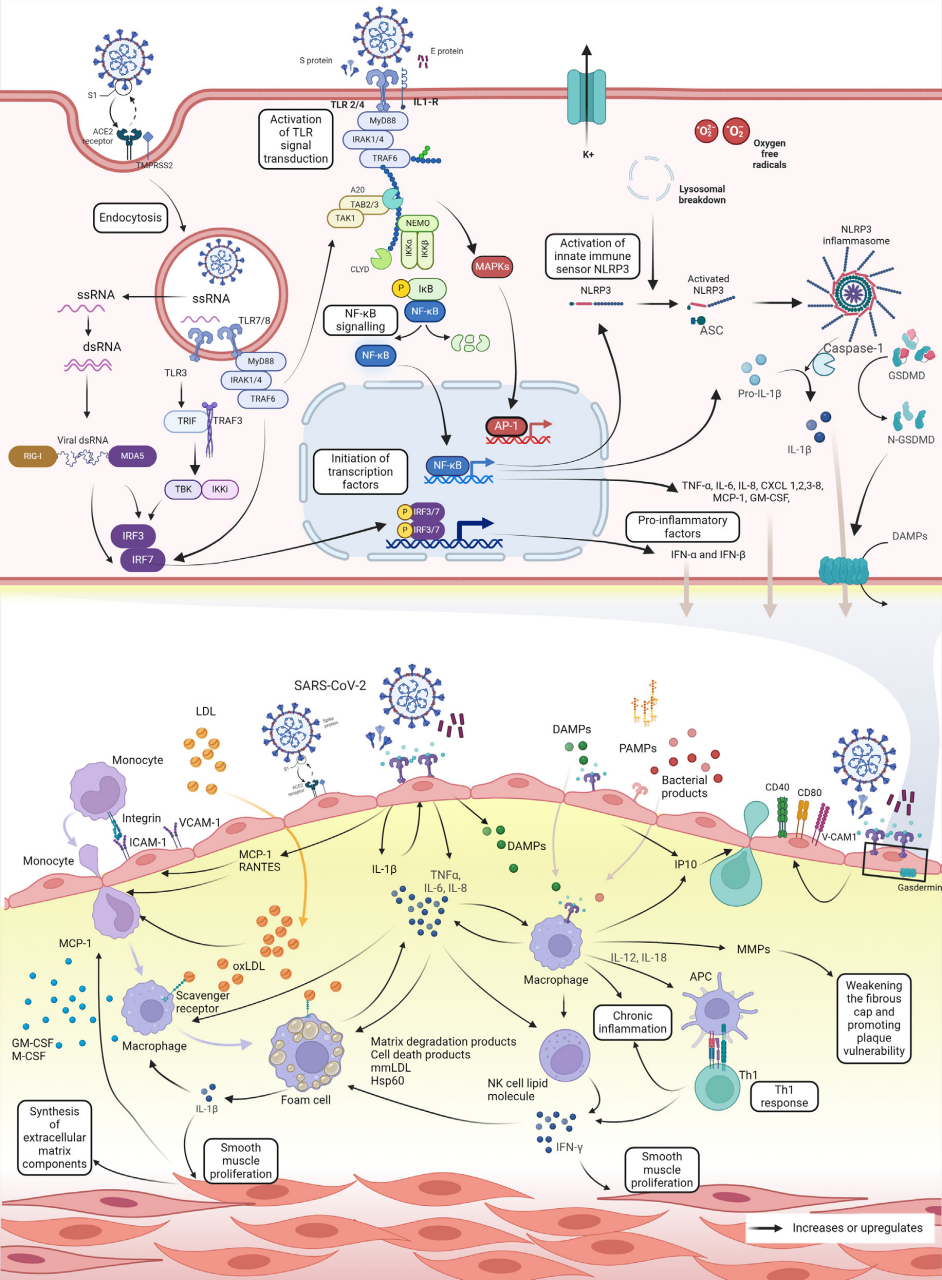
**Inflammatory and Immune Mechanisms in the Association of COVID-19 and Atherosclerosis** Pro-inflammatory cytokines, IL-1β and TNF-α, Induce monocyte transmigration, differentiation into macrophages, and foam cell formation in atherosclerotic plaques. The Th1 response exacerbates atherosclerosis; IL-12 and IL-18 from macrophages promote Th1 Differentiation and IFN-γ secretion, which disrupts endothelial junctions, enhances monocyte infiltration, foam-cell formation, smooth muscle proliferation, and plaque destabilization. Image inset: activation of PRRs in SARS-CoV-2. SARS-CoV-2 spike protein binds to ACE2, is internalized into endosomes post-cathepsin-mediated cleavage, releasing viral RNA. Innate immune cells detect this through PRRs like TLRs, RLRs, and NLRs. Many viruses activate TLR signal transduction via MyD88, except TLR3, which uses TRIF, activating NF-κB, MAPKs, and IRF pathways. Normally, NF-κB is inactive, bound to IκB in the cytosol. In the canonical pathway, inflammatory stimuli activate the IκB Kinase (IKK) complex (IKKα, β, γ or NEMO), leading to IκB phosphorylation, ubiquitination, and degradation. This releases NF-κB to enter the nucleus and initiate transcription of target genes including CAMs, pro-inflammatory factors, including TNF-α, IL-6, and IL-1, as well as innate immune sensors, such as NLRP3. Created Using Biorender.com. ACE = angiotensin-converting enzyme; AP = activator protein; ASC = adaptor protein; DAMPs = damage-associated molecular patterns; dsRNA, double-stranded RNA; GSDMD = gasdermin D; ICAM = intercellular adhesion molecule; IFN = interferon; IKK = IκB kinase; IL = interleukin; IRAK1 = interleukin 1 receptor-associated kinase 1; IRF = IFN regulatory factors; MAPKs = mitogen-activated protein kinases; MCP-1 = monocyte chemoattractant protein-1; MDA-5 = anti-melanoma differentiation-associated gene 5; MyD88 = myeloid differentiation primary response 88; NF-κB = nuclear factor-κB; NLRs = NOD-like receptors; NLRP3 = Nod-like receptor family pyrin domain containing; PAMP = pathogen-associated molecular pattern; RLR = RIG I-like receptor; S = SARS-CoV-2 spike protein; ssRNA = single-stranded RNA; TBK1 = TANK-binding kinase 1 (TBK1); TF = tissue factor; TNF = tumor necrosis factor; TRAF1 = TNF receptor-associated factor 1; VCAM = vascular cell adhesion molecule.

The innate immune system is crucial in every step of SARS-CoV-2's interaction with host cells, influencing viral entry, association with PRRs, initiation of signaling pathways, and cytokine production. Figure 4 illustrates the inflammatory and immune mechanisms linking COVID-19 and atherosclerosis.

#### SARS-CoV-2 viral entry and PRR sensing

Upon SARS-CoV-2 S protein binding to the ACE2 receptor, the virus either releases its genomic RNA into the cytoplasm after viral-host membrane fusion^28^ or is internalized into endosomes after cathepsin-mediated cleavage.^95^ Key innate immune cells possess PRRs in the cell surface, endosomes, or cytoplasm to respond to PAMPs or DAMPs.^96^ PRRs relevant in COVID-19 include TLRs, RIG I-like receptors, and NOD-like receptors. Viruses often trigger TLR signaling via MyD88; however, TLR3 signals exclusively through TRIF, activating downstream NF-κB, mitogen-activated protein kinases, and IFN regulatory factors. Their nuclear translocation results in the transcriptional activation of pro-inflammatory cytokines (eg, TNF-α, IL-6, and IL-1) and innate immune sensors like NLRP3 (Figure 4).

TLR3 activation enables TRIF signaling, leading to IFN production.^97^ Studies highlight the role of TLR2 in innate immune activation in COVID-19,^98^ alongside TLR1, TLR4, and TLR6, with TLR4 demonstrating the highest affinity for the virus.^99^ Additionally, TLR7 and TLR8 are known to recognize antiphospholipid antibodies^100^ found in patients with severe COVID-19. Furthermore, RIG I-like receptors (MDA-5 and RIG-1), which are key IFN pathway regulators, sense the intracellular single-stranded RNA of SARS-CoV-2.^101^

#### Inflammasome activation

Inflammasomes, particularly NLRP3, are ring-like structures that assemble upon innate immune activation (Figure 4) and are pivotal in atherosclerosis by converting pro–IL-1β to IL-1β and enhancing expression of endothelial adhesion molecules (E-selectin, ICAM-1, and VCAM-1).^102^ Additionally, neutrophil extracellular traps, cholesterol crystals, ox-LDL cholesterol, and shear stress contribute to NLRP3 inflammasome assembly in atherosclerosis.^103^

SARS-CoV-2 activates NLRP3 inflammasome directly or indirectly, triggering cytokine (IL-1β and IL-18) release and activation of the pyroptosis pathway.^104^ Colchicine, an NLRP3 inflammasome inhibitor, has been found to improve outcomes in COVID-19 patients.^105^ Thus, NLRP3 inflammasome may amplify the inflammation associated with COVID-19, potentially accelerating the progression of atherosclerosis.

#### Cytokine signaling and cell death

Severe COVID-19 triggers a systemic inflammatory response, leading to multi-organ damage.^13^ Elevated cytokines (IL-1β, IL-6, TNF-α, IFN-γ, macrophage inflammatory protein 1α and 1β) and chemokines (CCL-2, CCL-3, and CCL-5) correlate with higher viral loads^106^ and worse COVID-19 prognosis.^13^^,^^106^ ECs, when exposed to pro-inflammatory cytokines, initiate transcriptional programs, inducing the expression of adhesion molecules and chemokines, promoting leukocyte recruitment and inflammation.^107^ This results in EC injury, increased vascular permeability, and end-organ damage.^108^ Through this amplification loop, ECs constitute a significant source of pro-inflammatory cytokines, characteristic of the cytokine storm in COVID-19.^108^ This excessive inflammation can persist due to pre-existing cardiometabolic risk factors and promote atherogenesis independent of hyperlipidemia.^109^

#### Potential therapies targeting key intermediates in inflammation

Canakinumab, an IL-1β neutralizing antibody, reduced major adverse cardiovascular events in the CANTOS trial,^110^ while anakinra, inhibiting both IL-1α and IL-1β, was shown to reduce inflammatory markers post-NSTEMI.^111^ Both agents improved the duration of hospital stay and short-term outcomes in patients with moderate-to-severe COVID-19.^112^^,^^113^ The utility of IL-1 inhibitors in COVID-19-related atherosclerosis warrants further investigation.

IL-6 inhibitors, like tocilizumab and ziltivekimab, have demonstrated cardiovascular benefits, including attenuated inflammatory response and decreased troponin release post-PCI in NSTEMI patients.^114^ They also showed improved myocardial salvage, as measured by magnetic resonance imaging, in patients with STEMI.^115^ Additionally, in severe COVID-19 patients, tocilizumab reduced short-term mortality as demonstrated in the REMAP-CAP^116^ and RECOVERY trials,^117^ underscoring its potential in acute inflammatory settings and possibly in COVID-19-related cardiovascular complications.

Colchicine, while not directly beneficial in the prognosis of COVID-19, has shown significant anti-inflammatory properties in cardiovascular settings. It downregulates E-selectin expression and decreases NLPR3 inflammasome activation, suppressing IL-1β and IL-6 release, which are critical inflammatory mechanisms in atherosclerosis.^118^ A meta-analysis revealed that low-dose colchicine reduced the risk of major adverse cardiovascular and the need for coronary revascularization across a broad spectrum of patients with coronary disease.^119^ Currently, no studies have evaluated targets in the inflammatory pathways regarding post-COVID-19 atherosclerosis. A comprehensive discussion of potential therapies is beyond the scope of this manuscript; readers are referred to more detailed reviews elsewhere.^120^^,^^121^

## Post-acute sequelae of COVID-19

Post-acute sequelae of COVID-19 (PASC), also called post-acute COVID-19 syndrome or long-COVID, involves sequelae 1 to 3 months after SARS-CoV-2 infection,^122^^,^^123^ with major cardiac symptoms including fatigue, dyspnea, chest pain, and palpitations.^124^^,^^125^ While several reports highlight myocarditis, postural orthostatic tachycardia syndrome, arrhythmias, and venous thromboembolism,^123^^,^^126^ there is growing attention to the long-term risk of subclinical vascular pathology and clinical coronary artery disease, as nearly one-third of PASC patients and half of cardiac referrals post-COVID-19 report chest pain.^124^^,^^125^ Although the true ASCVD burden post-acute COVID-19 remains undefined, emerging studies suggest an increased risk (Table 1). The influence of various SARS-CoV-2 variants, COVID-19 vaccination,^139^ and in-hospital therapies^140^ on the risk of developing ASCVD post-COVID-19 remains an area for future investigation. The pathogenic mechanisms implicated in long COVID-19-related ASCVD are detailed below and depicted in the Central Illustration.

**Table 1:** Studies Assessing the Risk of Atherosclerotic Cardiovascular Diseases (ASCVD) in Post-COVID-19 Patients

First Author, Year (Country)	Type of Study and Study Setting	Study Group	Comparison Group	Outcomes	Effect Size (95% CI)	Adjustment/Matching	Follow-Up Duration	Vaccination Information
Ziyad Al-Aly, 2021^127^ (USA)	Retrospective cohort National health care databases of the U.S. Department of Veterans Affairs	Nonhospitalized post-COVID-19 patients from VHA (N = 73,435)	Non-COVID-19 Control group (N = 4,990,835)	Myocardial infarction **(I21-I22)** Other acute coronary syndromes **(I24)** Angina (Chest pain) **(I20)** Stroke **(I63)**	HR: 1.04 (0.81-1.35) HR: 1.32 (1.30-1.46) HR: 1.61 (1.48-1.76) HR: 1.41 (1.18-1.69)	Severity of infection Demographic parameters	6 mo (at least 30 d after COVID-19 diagnosis)	Vaccination not reported.
Wang, 2022^128^ (USA)	Retrospective cohort U.S. Collaborative Network in TriNetX	Post-COVID-19 patients (N = 690,892)	Non-COVID-19 Control group (N = 690,892) (1:1 propensity score matched)	Myocardial infarction **(I21-I22)** Other acute coronary syndromes **(I24)** Angina (Chest pain) **(I20)** Stroke **(I63)**	HR: 1.83 (1.74-1.92) HR: 1.89 (1.74-2.10) HR: 1.27 (1.18-1.36) HR: 1.50 (1.45-1.55)	Propensity score matched for age, race, gender, SES, comorbidities, blood type, alcohol and nicotine dependence, BMI	12 mo (at least 30 d after COVID-19 diagnosis)	All patients were unvaccinated.
Xie, 2022^129^ (USA)	Retrospective cohort National health care databases of the U.S. Department of Veterans Affairs	Post-COVID-19 patients (N = 153,760)	Non-COVID-19 Control group (N = 5,637,647)	Myocardial infarction **(I21-I22)** Other acute coronary syndromes **(I24)** Angina (Chest pain) **(I20)** Stroke **(I63)**	HR: 1.63 (1.51-1.75) HR: 1.72 (1.56-1.90) HR: 1.52 (1.42-1.64) HR: 1.52 (1.43-1.62)	Adjusted for age, race, sex, ADI, BMI, smoking status, eGFR, systolic and diastolic blood pressure, comorbidities including cancer, CKD, chronic lung disease, dementia, diabetes, dysautonomia, hyperlipidemia, and hypertension, and health care use parameters, including the use number of outpatient and inpatient encounters and use of long-term care.	12 mo (at least 30 d after COVID-19 diagnosis)	∼0.2% patients vaccinated. These outcomes were not adjusted for vaccination.
Buckley, 2021^130^ (USA, Europe)	Retrospective cohort TriNetX	Post-COVID-19 with myocarditis (N = 17,910)	Post-COVID-19 Without myocarditis (n = 17,910) (1:1 Propensity- score matched)	Myocardial infarction	OR: 1.37 (1.17-1.61)	Propensity score matched for age, sex, race, and comorbidities, including hypertensive diseases, ischemic heart diseases, heart failure, cerebrovascular diseases, diabetes mellitus, CKD, chronic lung disease, diseases of the digestive and nervous systems.	6 months after COVID-19	Vaccination not reported.
Katsoularis, 2021^131^ (Sweden)	Retrospective matched cohort study SmiNet (Swedish Public Health Agency) database and the Swedish National Board of Health and Welfare register	Post-COVID-19 patients (N = 86,742)	Non-COVID-19 Control group (N = 348,481)	Myocardial infarction Stroke	OR: 3.41 (1.58-7.36) OR: 3.63 (1.69-7.80)	Controls matched for age, sex, and county of residence in Sweden	2 wk after COVID-19 diagnosis	Vaccination not reported.
Kim, 2022^132^ (Korea)	Retrospective cohort The Korean nationwide COVID-19 registry (on infection and vaccination) and the Korean National Health Insurance Service database	Post-COVID-19 Vaccinated 2 doses of mRNA vaccine (N = 168,310)	Post-COVID-19 Unvaccinated (N = 62,727)	Myocardial infarction Ischemic stroke	HR: 0.48 (0.25-0.94) HR: 0.40 (0.26-0.63)	Inverse probability of treatment weighting (IPTW) Age, sex, Charlson comorbidity index, hypertension, and insurance type	4 mo (at least 30 d after COVID-19 diagnosis)	Compared vaccinated and unvaccinated individuals as noted.
Knight, 2022^133^ (UK)	Retrospective cohort English and Welsh electronic health records	Hospitalized for COVID-19 (N = 125,985) Not hospitalized for COVID-19 (N = 1,319,789)	Non-COVID-19 Control group (N = 44,964,486)	Acute myocardial infarction Ischemic stroke Angina	HR: 1.75 (1.50-2.05) HR: 2.15 (1.88-2.47) HR: 1.53 (1.39-1.69)	Adjusted for age, sex, and region	27 to 49 wk after COVID-19 diagnosis	All patients were unvaccinated.
Wiemken, 2022^134^ (USA)	Retrospective cohort Nationwide health insurance claims data – U.S. Health Verity Real-Time Insights and Evidence database	Not requiring ICU for COVID-19 (44,385) Requiring ICU for COVID-19 (N = 21,069)	Outpatient treatment for COVID-19 (1,292,064)	Ischemic heart disease **(I20-I25)** Non-ICU vs outpatient ICU vs outpatient	HR: 1.24 (1.16-1.32) HR: 1.59 (1.45-1.74)	Inverse probability of treatment weighting (IPTW) to account for imbalances in baseline characteristics	9 mo (at least 30 d after COVID-19 diagnosis)	Receipt of at least 1 dose of COVID-19 vaccine was accounted for in the propensity score.
Wan, 2023^135^ (UK)	Retrospective cohort The UK Biobank	Post-COVID-19 patients (N = 7,584)	Contemporary non-COVID-19 Control group (N = 75,790)	CHD Myocardial infarction Other acute coronary syndromes Stroke	HR: 5.0 (2.8-8.7) HR: 2.7 (1.1-7.2) HR: 4.3 (1.2-15.7) HR: 9.7 (3.8-24.9)	Adjusted for age, sex, smoking, diabetes mellitus, hypertension, Charlson comorbidity index, baseline BMI, ethnicity, index of multiple deprivation, and history of outcome measures before weighing.	18 mo (at least 21 d after COVID-19 diagnosis)	All patients were unvaccinated.
Raisi-Estabragh, 2023^136^ (UK)	Retrospective cohort The UK Biobank	Post COVID-19 patients (N = 17,871)	Contemporary Non-COVID-19 Control group (N = 35,742)	Myocardial infarction (All COVID-19) Myocardial infarction (hospitalized due to COVID-19)	HR: 9.9 (3.4-29.1) HR: 22.2 (2.8-173)	Propensity score matched for age, sex, Townsend score (deprivation), BMI, ethnicity, diabetes, prevalent ischemic heart disease (IHD), smoking, hypertension, and high cholesterol.	5 mo (at least 30 d after COVID-19 diagnosis)	Vaccination not reported.
DeVries, 2023^137^ (USA)	Retrospective cohort national insurance claims data enhanced with data from the Social Security Administration’s death master file.	Post-COVID-19 patients (N = 13,435)	Non-COVID-19 Control group (N = 26,870)	Coronary artery disease Ischemic stroke	RR: 1.78 (1.70-1.88) RR: 2.17 (1.98-2.52)	Propensity score matched for age, sex, region, race, ethnicity, education, socioeconomic status, Elixhauser Comorbidity Index, comorbid conditions, and 6-mo baseline health care utilization.	12 mo (at least 30 d after COVID-19 diagnosis)	All patients were unvaccinated.
Koyama, 2023^138^ (USA)	Retrospective cohort IQVIA PharMetrics Plus insurance claims database	Post-COVID-19 patients (N = 2,983,857)	Non-COVID-19 Control group (N = 22,582,479)	Ischemic heart disease (without diabetes) Ischemic heart disease (with diabetes) (composite of myocardial infarction, other acute coronary syndromes, ischemic cardiomyopathy, angina)	HR: 1.68 (1.62-1.74) HR: 1.71 (1.62-1.80)	Adjusted for age, sex, private health insurance, U.S. Census division, Charlson comorbidity index, and any pre-existing cardiovascular condition.	Mean 8.5 mo (at least 30 d after COVID-19 diagnosis)	Vaccination not reported.

BMI = body mass index; CHD = coronary heart disease; CI = confidence interval; CKD = chronic kidney disease; eGFR = estimated glomerular filtration rate; ICU = intensive care unit; IPTW = inverse probability of treatment weighting; SES = socioeconomic status.

**Central illustration:**
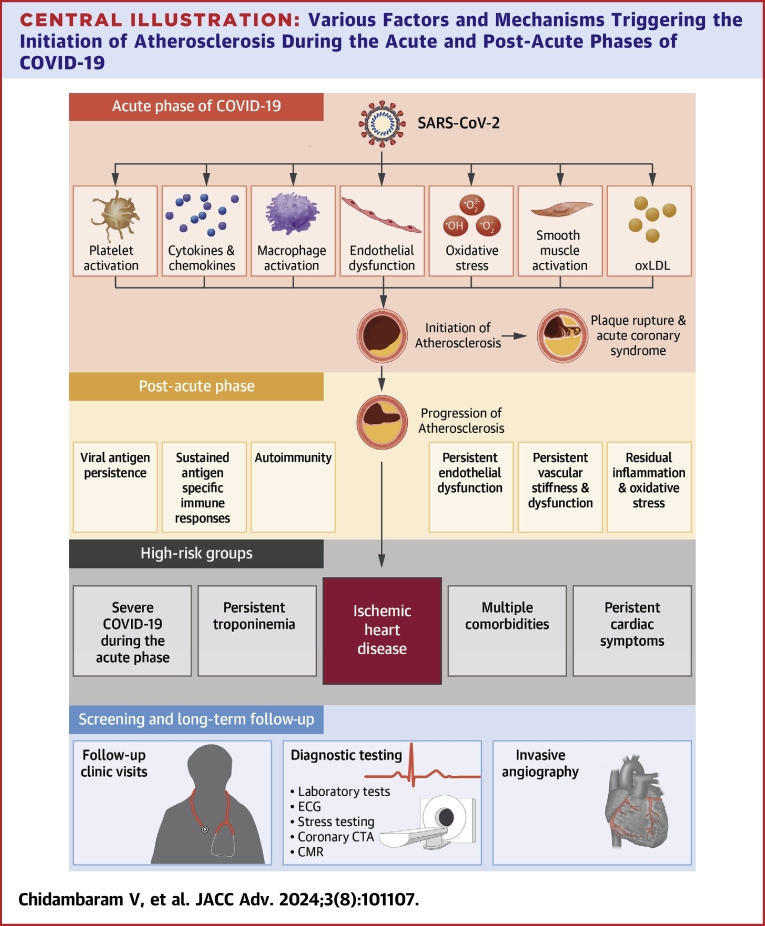
**Various Factors and Mechanisms Triggering the Initiation of Atherosclerosis During the Acute and Post-Acute Phases of COVID-19** High-risk groups need to be identified for screening and long-term follow-up after COVID-19. CMR = cardiac magnetic resonance; CTA = computed tomographic angiography; ECG = electrocardiogram.

### Pathogenic mechanisms in PASC

#### Viral antigen persistence

Besides infectious particles in airways during acute COVID-19, viral RNA and antigens are detected in the central nervous system and lymphoid organs,^141^ as well as in the feces for months.^142^ However, these RNA or antigen reservoirs^143^ in PASC do not appear to reflect persistent or latent viral infection, as the virus cannot be cultured from these sources.^142^ Peluso et al demonstrated circulating SARS-CoV-2 S protein in PASC patients 12 months post-diagnosis, but N protein less frequently, arguing against active viral reservoirs.^144^

#### Immune mechanisms and dysregulation

Postinfection, most patients generate long-lasting SARS-CoV-2-specific CD4^+^ and CD8^+^ T-cell and B-cell responses,^145^ often increasing over time,^145^^,^^146^ which are independent of COVID-19 vaccination status^147^ and unrelated to prolonged viral replication or *de-novo* antigen production.^148^ At 6 months, S-specific CD4+ TCR clonal depth correlated with COVID-19 severity and long COVID symptoms,^149^ while CD8^+^ T-cell responses correlated with pre-existing lung disease. Follicular dendritic cells retain SARS-CoV-2 antigens in germinal centers for months, driving memory B-cell maturation,^146^ with unique immunoglobulin signatures in PASC patients.^150^ Vaccinated individuals showed reduced risk^151^ and earlier resolution of long COVID-19 symptoms,^152^ possibly due to faster return to immunological baseline or antigen clearance.

In acute COVID-19, T cells and NK cells decline due to SARS-CoV-2-induced apoptosis.^153^ However, 9 to 12 months postinfection, PASC patients exhibited increased CD4+ and CD8+ effector T cells, Th9 cells, and naive B cells,^154^ arguing against sustained T-cell dysfunction.^147^ After infection, heightened Th9^154^ and Th17 CD4+ subsets emerged,^155^ opposing the induction of Treg cells, which protect against atherogenesis. Convalescent subjects displayed CD69+ CD103- CD8+ T cells expressing EOMES, granzyme B, and granzyme K, which are linked to fibroblast activation and advanced atherosclerosis.^156^

#### Autoimmunity

Severe COVID-19 and PASC may accompany persistent, general, or tissue-specific^157^ (blood vessels, heart, or brain) autoimmune responses, likely secondary to transient loss of self-tolerance or inappropriate immune reconstitution.^154^ Initially thought to sustain viral reservoirs, anti-IFN type I antibodies do not contribute to PASC.^158^ Autoantibodies associated with atherosclerosis, like antinuclear antibodies, ACA (anti-cardiolipin antibody), and β2GP1 autoantibodies, were observed in PASC patients,^159^^,^^160^ suggesting possible autoimmunity following acute COVID-19. However, there is no conclusive evidence regarding the significance of SARS-CoV-2-directed antibodies in relation to long COVID-19,^161^ and it remains a subject of ongoing investigation.

#### Persistent endothelial and vascular dysfunction

EC activation persists months after SARS-CoV-2 infection.^162^ Abnormal flow-mediated dilation, a noninvasive test reflecting impaired endothelial function,^163^ indicates subclinical atherosclerosis^164^ and predicts ASCVD events.^165^ In post-COVID-19 patients, brachial artery flow-mediated dilation and carotid-femoral pulse wave velocity^166^ demonstrated reduced vascular function compared to controls (Table 2). Verma et al noted lower myocardial flow reserve and impaired epicardial vasodilation in PASC patients.^178^ Excessive ROS production in COVID-19 may perpetuate long-term EC dysfunction.^179^ Circulating ECs, a biomarker of vascular injury, are elevated in patients with acute COVID-19^180^ and during convalescence compared to healthy controls.^181^

**Table 2:** Studies Evaluating Endothelial and Vascular Function in Post-COVID-19 Patients

First Author, Year (Country)	Type of Study	Study Group	Control Number of Patients	Outcome	Statistic	Adjusted for	Follow-Up Duration
Ambrosino, 2021^167^ (Italy)	Matched prospective cohort study	Post-COVID-19 patients (N = 133)	Non-COVID-19 Control group (N = 133) (1:1 matched)	Brachial artery flow-mediated dilatation (FMD)	3.2% ± 2.6 vs 6.4% ± 4.1 (*P* < 0.001)	Age, gender, and cardiovascular risk factors	2 mo after COVID-19 diagnosis
Oikonomou, 2022^168^ (Greece)	Matched prospective cohort study	Post-COVID-19 patients (N = 73) ICU (N = 46) vs Non-ICU (N = 27)	Non-COVID-19 historical control group (N = 73) (1:1 propensity- score matched)	Brachial artery flow-mediated dilatation (FMD)	5.2% ± 1.6 vs 6.5% ± 3.1 (*P* = 0.01) (ICU vs non-ICU) 3.2% ± 0.7 vs 5.7% ± 1.4 (*P* < 0.001)	Age, sex, current smoking, arterial hypertension, diabetes mellitus, dyslipidemia, and coronary artery disease	6 mo after COVID-19 diagnosis
Riou, 2021^169^	Prospective cohort study	Post-COVID-19 patients (N = 27)	Non-COVID-19 Control group (N = 9) (age and sex matched)	Brachial artery flow-mediated dilatation (FMD)	8.2 (IQR: 7.2-8.9) vs 10.3 (IQR: 9.1-1.7) *P* = 0.002	Age and sex	3 mo after COVID-19 diagnosis
Santoro, 2022^170^	Prospective cohort study	Post-COVID-19 patients: required hospitalization (N = 303) No oxygen group (N = 127) Oxygen group (N = 115) Invasive ventilation group (N = 61)	Post-COVID-19 patients: Outpatient treatment (N = 382)	Brachial artery flow-mediated dilatation (FMD)	Outpatient treatment 12.0 ± 4.3 No oxygen group 10.6 ± 4.7 Oxygen group 10.3 ± 4.6 Invasive ventilation 9.4 ± 4.3	Age, sex, BMI, arterial hypertension, diabetes mellitus, and CRP levels	3 mo after COVID-19 diagnosis
Nandadeva, 2021^171^	Prospective cohort study	Post-COVID-19 patients (N = 16) Symptomatic (N = 8) Asymptomatic (N = 8)	Non-COVID-19 Control group (N = 23)	Brachial artery flow-mediated dilation (FMD)	Symptomatic 3.8% ± 0.6 vs Asymptomatic 6.8% ± 0.9 (*P* = 0.007) vs Control 6.8% ± 0.6 (*P* = 0.003)	NA	4 wk after COVID-19 diagnosis
Ratchford, 2020^166^	Cross-sectional study	Post-COVID-19 patients (N = 11)	Non-COVID-19 Control group (N = 20)	Brachial artery flow-mediated dilatation (FMD) Carotid-femoral pulse wave velocity (PWVcf)	2.71% ± 1.21 vs 8.81% ± 2.96 (*P* < 0.01) Mean (SD) 5.83 ± 0.62 m/s vs 5.17 ± 0.66 m/s (*P* < 0.01).	NA	3-4 wk after COVID-19 diagnosis
Szeghy, 2022^172^	Cross-sectional study	Post-COVID-19 patients (N = 15)	Non-COVID-19 Control group (N = 15)	Carotid-femoral pulse wave velocity (PWVcf)	6.0 ± 1.0 m/s vs 5.0 ± 1.0 m/s (*P* = 0.02)	Height, weight, physical activity, and contraceptive use	3-4 wk after COVID-19 diagnosis
Zanoli, 2022^173^	Cross-sectional study	Post-COVID-19 patients (N = 90)	Non-COVID-19 Control group (N = 180)	Aortic pulse wave velocity (aPWV) (m/s) Brachial pulse wave velocity (aPWV) (m/s)	9.0 ± 2.4 vs 7.9 ± 1.5 (*P* < 0.05) 7.3 ± 1.4 vs 6.3 ± 1.1 (*P* < 0.05)	Age, sex, BMI mean blood pressure, eGFR, and total cholesterol	6 mo after COVID-19 diagnosis
Weber, 2022^174^	Retrospective cohort study	Post-COVID-19 patients (N = 34)	Non-COVID-19 Control group (N = 103)	Myocardial blood flow reserve (mL/min/g) stress myocardial perfusion PET imaging Abnormal myocardial blood flow reserve (<2 mL/min/g)	2.00 ± 0.45 vs 2.48 ± 0.47 (*P* < 0.0 01) Proportion 44.0% vs 11.7% (*P* < 0.001)	Control group matched for age, sex, diabetes, obesity, hyperlipidemia, hypertension, and history of coronary artery disease	4.6 mo (median) after COVID-19 diagnosis
Tong, 2022^175^	Prospective cohort study	Post-COVID-19 patients (N = 345)	Non-COVID-19 Control group (N = 119)	VCAM-1 ICAM-1	Median 1.69 vs 1.67 ng/mL (*P* = 0.363) Median 427.3 vs 469.7 pg/mL, (*P* = 0.139)	Age and gender matched	1 y after COVID-19 diagnosis
Charfeddine, 2021^176^	Prospective cohort study	Patients with long COVID-19 symptoms	Patients without long COVID-19 symptoms	Endothelial quality index (EQI)	OR for EQI <2 for long COVID-19 syndrome 1.52 (1.07-2.16)	Age, sex, diabetes, hypertension, dyslipidemia, coronary heart disease, and severity of acute COVID-19	6 mo after COVID-19 diagnosis
Poyatos, 2022^177^	Prospective cohort study	Post-COVID-19 patients (N = 32)	Non-COVID-19 Control group (N = 31)	Endothelial colony forming cells (ECFCs)	2.81 ± 2.33 vs 1.23 ± 1.86 (*P* = 0.001)	NA	3 mo after COVID-19 diagnosis

aPWV = aortic pulse wave velocity; BMI = body mass index; CKD = chronic kidney disease; ECFCs = endothelial colony forming cells; eGFR = estimated glomerular filtration rate; EQI = Endothelial quality index; FMD = flow-mediated dilatation; ICAM = intercellular adhesion molecule; ICU = intensive care unit; IPTW = inverse probability of treatment weighting; PWVcf = carotid-femoral pulse wave velocity; SES = socioeconomic status; VCAM = vascular cell adhesion molecule.

#### Residual inflammation

Dysregulated inflammation^182^ accompanies PASC, with elevated levels of pro-inflammatory cytokines (IL-1β, IL-6, TNF-α, and IFN-γ) and chemokines (CXCL9 and CXCL10).^154^^,^^182^^,^^183^ Phetsouphanh et al described persistent innate immune activation months after nonsevere SARS-CoV-2 infection.^183^ [^18^F]fluorodeoxyglucose positron emission tomography/computed tomography identified sustained inflammation in select vascular regions 30 days post-COVID-19.^184^ This unresolved vascular inflammation^185^ may drive EC dysfunction and effector lymphocyte activation.^181^

### Risk factors and risk stratification for ASCVD for post-COVID-19

Patients post-COVID-19, even those with previously low cardiac risk, demonstrate a higher risk of ASCVD.^129^ This underscores the need to regard COVID-19, especially severe illness, as a risk factor for atherosclerosis. The risk factors for PASC include increased age, female sex, comorbidities (obesity, diabetes, and hypertension), acute COVID-19 severity, intensive care unit admission,^186^ prolonged hospitalization, SARS-CoV-2 viremia, and elevated inflammatory (ferritin and C-reactive protein) and cardiac (troponin and B-type natriuretic peptide) biomarkers.^187^ Given the magnitude of patients affected by COVID-19, identifying high-risk groups is important for cost-effective coronary artery disease (CAD) evaluation in PASC patients with cardiac symptoms, especially those with exertional chest pain.

### Further cardiac testing in select patients

#### Importance in patients with elevated risk

Despite the growing body of research, there remains a notable lack of definitive evidence to guide specific screening strategies for ASCVD in patients with PASC. An urgent need exists for a systematic decision pathway for cardiac evaluation in these individuals, utilizing noninvasive cardiac testing to: 1) further improve cardiac risk stratification and enhance patient dialogue; 2) identify patients requiring prompt and aggressive pharmacological preventive therapies (statins, aspirin, or antihypertensives); 3) enable early identification of obstructive CAD; and 4) optimize prognosis by reducing major cardiac complications, such as MI, heart failure, and sudden cardiac death. In the absence of enough available evidence, we recommend a proactive and meticulous approach to CV screening in the high-risk post-COVID-19 population; particularly those over 65 years of age, individuals with comorbidities such as diabetes or hypertension, persistent troponinemia, ongoing cardiac symptoms, and those who experienced severe COVID-19 necessitating intensive care unit admission or prolonged hospitalization.

#### Pretest probability and selection of cardiac test

The choice of noninvasive cardiac evaluation, either functional (stress echocardiogram, single-photon emission computed tomography, cardiac magnetic resonance or positron emission tomography—computed tomography) or anatomical (coronary computed tomography angiography), depends on patient-related factors, pretest probability of significant CAD, symptom severity, and testing constraints.^188^ Patients requiring hospital admission for COVID-19 had a 3 to 4 times higher risk of MI in the 6 months postdischarge compared to outpatients.^189^ Whether currently available pretest probability scores for assessing obstructive CAD^190^ in patients with acute or stable chest pain have good discriminatory power and whether early invasive strategy is useful in post-COVID-19 patients, especially after intensive care unit admission, when compared to non-COVID-19 patients is currently unknown. Thus, further studies are needed to develop personalized risk prediction algorithms for obstructive CAD in patients following acute COVID-19 and to identify appropriate diagnostic tests and primary and secondary treatment strategies.

## Conclusions

With increasing awareness and accumulating evidence regarding cardiovascular involvement during the acute and chronic phases of COVID-19, an improved understanding of the overlap between the pathogenesis of SARS-CoV-2 and ASCVD could have actionable implications and allow for novel interventions directed at various steps in the pathogenesis. Additionally, the identification of cost-effective screening and follow-up strategies across diverse risk groups will have significance beyond COVID-19 and enable the management of cardiovascular diseases in patients with other chronic infections.

## Funding support and author disclosures

This study was supported by the 10.13039/100006492National Institute of Allergy and Infectious Diseases (NIAID)/10.13039/100000002National Institutes of Health (NIH) grant K24AI143447 to Dr Karakousis. All other authors have reported that they have no relationships relevant to the contents of this paper to disclose.

## References

[1] Dai H., Much A.A., Maor E. (2020). Global, regional, and national burden of ischaemic heart disease and its attributable risk factors, 1990–2017: results from the Global Burden of Disease Study 2017. Eur Heart J Qual Care Clin Outcomes.

[2] Anon Cardiovascular diseases (CVDs). https://www.who.int/en/news-room/fact-sheets/detail/cardiovascular-diseases.

[3] Libby P., Ridker P.M., Hansson G.K. (2011). Progress and challenges in translating the biology of atherosclerosis. Nature.

[4] Post W.S., Budoff M., Kingsley L. (2014). Associations between HIV infection and subclinical coronary atherosclerosis. Ann Intern Med.

[5] Huaman M.A., de Cecco C.N., Bittencourt M.S. (2021). Latent Tuberculosis infection and subclinical coronary atherosclerosis in Peru and Uganda. Clin Infect Dis.

[6] Galkina E., Ley K. (2009). Immune and inflammatory mechanisms of atherosclerosis. Circ Res.

[7] Msemburi W., Karlinsky A., Knutson V., Aleshin-Guendel S., Chatterji S., Wakefield J. (2022). The WHO estimates of excess mortality associated with the COVID-19 pandemic. Nature.

[8] Jin Y., Ji W., Yang H., Chen S., Zhang W., Duan G. (2020). Endothelial activation and dysfunction in COVID-19: from basic mechanisms to potential therapeutic approaches. Signal Transduct Target Ther.

[9] Kanth M.B., Denorme F., Middleton E.A. (2020). Platelet gene expression and function in patients with COVID-19. Blood.

[10] Naghavi M., Wyde P., Litovsky S. (2003). Influenza infection exerts prominent inflammatory and thrombotic effects on the atherosclerotic plaques of apolipoprotein E-deficient mice. Circulation.

[11] Davis H.E., McCorkell L., Vogel J.M., Topol E.J. (2023). Long COVID: major findings, mechanisms and recommendations. Nat Rev Microbiol.

[12] Zhou F., Yu T., Du R. (2020). Clinical course and risk factors for mortality of adult inpatients with COVID-19 in Wuhan, China: a retrospective cohort study. Lancet.

[13] Chidambaram V., Tun N.L., Haque W.Z. (2020). Factors associated with disease severity and mortality among patients with COVID-19: a systematic review and meta-analysis Bhatt GC. PLoS One.

[14] Bangalore S., Sharma A., Slotwiner A. (2020). ST-segment elevation in patients with covid-19 - a case series. N Engl J Med.

[15] Bavishi C., Bonow R.O., Trivedi V., Abbott J.D., Messerli F.H., Bhatt D.L. (2020). Special Article - acute myocardial injury in patients hospitalized with COVID-19 infection: a review. Prog Cardiovasc Dis.

[16] Basso C., Leone O., Rizzo S. (2020). Pathological features of COVID-19-associated myocardial injury: a multicentre cardiovascular pathology study. Eur Heart J.

[17] Fox S.E., Akmatbekov A., Harbert J.L., Li G., Quincy Brown J., Vander Heide R.S. (2020). Pulmonary and cardiac pathology in African American patients with COVID-19: an autopsy series from New Orleans. Lancet Respir Med.

[18] Almamlouk R., Kashour T., Obeidat S. (2022). COVID-19–Associated cardiac pathology at the postmortem evaluation: a collaborative systematic review. Clin Microbiol Infect.

[19] Stefanini G.G., Montorfano M., Trabattoni D. (2020). ST-Elevation myocardial infarction in patients with COVID-19: clinical and angiographic outcomes. Circulation.

[20] Garcia S., Dehghani P., Grines C. (2021). Initial findings from the North American COVID-19 myocardial infarction registry. J Am Coll Cardiol.

[21] Choudry F.A., Hamshere S.M., Rathod K.S. (2020). High thrombus burden in patients with COVID-19 presenting with ST-segment elevation myocardial infarction. J Am Coll Cardiol.

[22] Masi P., Hékimian G., Lejeune M. (2020). Systemic inflammatory response syndrome is a major contributor to COVID-19-associated coagulopathy: insights from a prospective, single-center cohort study. Circulation.

[23] Alexander Y., Osto E., Schmidt-Trucksäss A. (2021). Endothelial function in cardiovascular medicine: a consensus paper of the European Society of Cardiology Working groups on atherosclerosis and vascular biology, Aorta and peripheral vascular diseases, coronary pathophysiology and Microcirculation, and thrombosis. Cardiovasc Res.

[24] Gimbrone M.A., García-Cardeña G. (2016). Endothelial cell dysfunction and the Pathobiology of atherosclerosis. Circ Res.

[25] Pober J.S., Sessa W.C. (2007). Evolving functions of endothelial cells in inflammation. Nat Rev Immunol.

[26] Gerhardt T., Ley K. (2015). Monocyte trafficking across the vessel wall. Cardiovasc Res.

[27] Monteil V., Kwon H., Prado P. (2020). Inhibition of SARS-CoV-2 infections in Engineered human tissues using clinical-Grade soluble human ACE2. Cell.

[28] Hoffmann M., Kleine-Weber H., Schroeder S. (2020). SARS-CoV-2 cell entry depends on ACE2 and TMPRSS2 and is Blocked by a clinically proven protease inhibitor. Cell.

[29] Casciola-Rosen L., Thiemann D.R., Andrade F. (2022). IgM anti-ACE2 autoantibodies in severe COVID-19 activate complement and perturb vascular endothelial function. JCI Insight.

[30] Varga Z., Flammer A.J., Steiger P. (2020). Endothelial cell infection and endotheliitis in COVID-19. Lancet.

[31] Goldsmith C.S., Miller S.E., Martines R.B., Bullock H.A., Zaki S.R. (2020). Electron microscopy of SARS-CoV-2: a challenging task. Lancet.

[32] Carsana L., Sonzogni A., Nasr A. (2020). Pulmonary postmortem findings in a series of COVID-19 cases from northern Italy: a two-centre descriptive study. Lancet Infect Dis.

[33] Yang J.M., Dong M., Meng X. (2013). Angiotensin-(1-7) dose-dependently inhibits atherosclerotic lesion formation and enhances plaque stability by targeting vascular cells. Arterioscler Thromb Vasc Biol.

[34] Gwathmey T.M., Pendergrass K.D., Reid S.D., Rose J.C., Diz D.I., Chappell M.C. (2010). Angiotensin-(1-7)-ACE2 attenuates reactive oxygen species formation to Angiotensin II within the cell nucleus. Hypertension.

[35] Wang J., Zhao H., An Y. (2021). ACE2 shedding and the role in COVID-19. Front Cell Infect Microbiol.

[36] Soro-Paavonen A., Gordin D., Forsblom C. (2012). Circulating ACE2 activity is increased in patients with type 1 diabetes and vascular complications. J Hypertens.

[37] Nicin L., Abplanalp W.T., Mellentin H. (2020). Cell type-specific expression of the putative SARS-CoV-2 receptor ACE2 in human hearts. Eur Heart J.

[38] McCracken I.R., Saginc G., He L. (2021). Lack of evidence of angiotensin-converting enzyme 2 expression and replicative infection by SARS-CoV-2 in human endothelial cells. Circulation.

[39] Wagner J.U.G., Bojkova D., Shumliakivska M. (2021). Increased susceptibility of human endothelial cells to infections by SARS-CoV-2 variants. Basic Res Cardiol.

[40] Wei C., Wan L., Yan Q. (2020). HDL-scavenger receptor B type 1 facilitates SARS-CoV-2 entry. Nat Metab.

[41] Schimmel L., Chew K.Y., Stocks C.J. (2021). Endothelial cells are not productively infected by SARS-CoV-2. Clin Transl Immunol.

[42] Mancia G., Rea F., Ludergnani M., Apolone G., Corrao G. (2020). Renin–angiotensin–aldosterone system Blockers and the risk of covid-19. N Engl J Med.

[43] Cantuti-Castelvetri L., Ojha R., Pedro L.D. (2020). Neuropilin-1 facilitates SARS-CoV-2 cell entry and infectivity. Science.

[44] Wang K., Chen W., Zhang Z. (2020). CD147-spike protein is a novel route for SARS-CoV-2 infection to host cells. Signal Transduct Target Ther.

[45] Rauch A., Dupont A., Goutay J. (2020). Endotheliopathy is induced by plasma from critically Ill patients and associated with organ failure in severe COVID-19. Circulation.

[46] Hajra L., Evans A.I., Chen M., Hyduk S.J., Collins T., Cybulsky M.I. (2000). The NF-κB signal transduction pathway in aortic endothelial cells is primed for activation in regions predisposed to atherosclerotic lesion formation. Proc Natl Acad Sci U S A.

[47] Cheng W., Cui C., Liu G. (2022). NF-κB, A potential therapeutic target in cardiovascular diseases. Cardiovasc Drugs Ther.

[48] Flory E., Kunz M., Scheller C. (2000). Influenza virus-induced NF-κB-dependent gene expression is mediated by Overexpression of viral proteins and involves oxidative Radicals and activation of IκB kinase. J Biol Chem.

[49] Schreck R., Rieber P., Baeuerle P.A. (1991). Reactive oxygen intermediates as apparently widely used messengers in the activation of the NF-kappa B transcription factor and HIV-1. EMBO J.

[50] Sun S.-C., Ballard D.W. (1999). Persistent activation of NF-κB by the Tax transforming protein of HTLV-1: hijacking cellular IκB kinases. Oncogene.

[51] Fratta Pasini A.M., Stranieri C., Cominacini L., Mozzini C. (2021). Potential role of Antioxidant and anti-inflammatory therapies to prevent severe SARS-Cov-2 complications. Antioxidants.

[52] Robles J.P., Zamora M., Adan-Castro E., Siqueiros-Marquez L., de la Escalera G.M., Clapp C. (2022). The spike protein of SARS-CoV-2 induces endothelial inflammation through integrin α5β1 and NF-κB signaling. J Biol Chem.

[53] Wu Y., Ma L., Cai S. (2021). RNA-induced liquid phase separation of SARS-CoV-2 nucleocapsid protein facilitates NF-κB hyper-activation and inflammation. Signal Transduct Target Ther.

[54] Ma Z., Li X., Fan R.L.Y. (2022). A human pluripotent stem cell-based model of SARS-CoV-2 infection reveals an ACE2-independent inflammatory activation of vascular endothelial cells through TLR4. Stem Cell Rep.

[55] Khan S., Shafiei M.S., Longoria C., Schoggins J.W., Savani R.C., Zaki H. (2021). SARS-CoV-2 spike protein induces inflammation via TLR2-dependent activation of the NF-κB pathway. Elife.

[56] Gudowska-Sawczuk M., Mroczko B. (2022). The role of nuclear factor kappa B (NF-κB) in development and treatment of COVID-19: review. Int J Mol Sci.

[57] Cook-Mills J.M., Marchese M.E., Abdala-Valencia H. (2011). Vascular cell adhesion molecule-1 expression and signaling during disease: regulation by reactive oxygen species and Antioxidants. Antioxid Redox Signal.

[58] Wee H., Oh H.M., Jo J.H., Jun C.D. (2009). ICAM-1/LFA-1 interaction contributes to the induction of endothelial cell-cell separation: implication for enhanced leukocyte diapedesis. Exp Mol Med.

[59] Rohde L.E., Richard T.L., Rivero J. (1998). Circulating cell adhesion molecules are correlated with Ultrasound-based assessment of carotid atherosclerosis. Arterioscler Thromb Vasc Biol.

[60] Yao S., Luo N., Liu J. (2021). Elevated Serum levels of Progranulin and soluble vascular cell adhesion molecule-1 in patients with COVID-19. J Inflamm Res.

[61] Birnhuber A., Fließer E., Gorkiewicz G. (2021). Between inflammation and thrombosis: endothelial cells in COVID-19. Eur Respir J.

[62] Rotoli B.M., Barilli A., Visigalli R., Ferrari F., Dall'Asta V. (2021). Endothelial cell activation by SARS-CoV-2 spike S1 protein: a Crosstalk between endothelium and innate immune cells. Biomedicines.

[63] Tong M., Yan X., Jiang Y. (2022). Endothelial biomarkers in patients recovered from COVID-19 one Year after hospital discharge: a Cross-Sectional study. Mediterr J Hematol Infect Dis.

[64] Fan B.E., Wong S.W., Sum C.L.L. (2022). Hypercoagulability, endotheliopathy, and inflammation approximating 1 year after recovery: assessing the long-term outcomes in COVID-19 patients. Am J Hematol.

[65] Li H., Horke S., Förstermann U. (2014). Vascular oxidative stress, nitric oxide and atherosclerosis. Atherosclerosis.

[66] Lubos E., Kelly N.J., Oldebeken S.R. (2011). Glutathione peroxidase-1 deficiency augments proinflammatory cytokine-induced redox signaling and human endothelial cell activation. J Biol Chem.

[67] Costa T.J., Potje S.R., Fraga-Silva T.F.C. (2022). Mitochondrial DNA and TLR9 activation contribute to SARS-CoV-2-induced endothelial cell damage. Vascul Pharmacol.

[68] Montiel V., Lobysheva I., Gérard L. (2022). Oxidative stress-induced endothelial dysfunction and decreased vascular nitric oxide in COVID-19 patients. EBioMedicine.

[69] Mehta P.K., Griendling K.K. (2007). Angiotensin II cell signaling: physiological and pathological effects in the cardiovascular system. Am J Physiol Cell Physiol.

[70] Lindemann S., Krämer B., Daub K., Stellos K., Gawaz M. (2007). Molecular pathways used by platelets to initiate and accelerate atherogenesis. Curr Opin Lipidol.

[71] Huo Y., Schober A., Forlow S.B. (2003). Circulating activated platelets exacerbate atherosclerosis in mice deficient in apolipoprotein E. Nat Med.

[72] Zaid Y., Puhm F., Allaeys I. (2020). Platelets can associate with SARS-CoV-2 RNA and are hyperactivated in COVID-19. Circ Res.

[73] Hottz E.D., Azevedo-Quintanilha I.G., Palhinha L. (2020). Platelet activation and platelet-monocyte aggregate formation trigger tissue factor expression in patients with severe COVID-19. Blood.

[74] Garcia C., Duong J.A., Po M. (2022). Platelet activation and partial desensitization are associated with viral xenophagy in patients with severe COVID-19. Blood Adv.

[75] Zhang S., Liu Y., Wang X. (2020). SARS-CoV-2 binds platelet ACE2 to enhance thrombosis in COVID-19. J Hematol Oncol.

[76] Shen S., Zhang J., Fang Y. (2021). SARS-CoV-2 interacts with platelets and megakaryocytes via ACE2-independent mechanism. J Hematol Oncol.

[77] Li T., Yang Y., Li Y. (2022). Platelets mediate inflammatory monocyte activation by SARS-CoV-2 spike protein. J Clin Invest.

[78] Koupenova M. (2020). Potential role of platelets in COVID-19: implications for thrombosis. Res Pract Thromb Haemost.

[79] Koupenova M., Corkrey H.A., Vitseva O. (2021). SARS-CoV-2 initiates programmed cell death in platelets. Circ Res.

[80] Puhm F., Allaeys I., Lacasse E. (2022). Platelet activation by SARS-CoV-2 implicates the release of active tissue factor by infected cells. Blood Adv.

[81] Guervilly C., Bonifay A., Burtey S. (2021). Dissemination of extreme levels of extracellular vesicles: tissue factor activity in patients with severe COVID-19. Blood Adv.

[82] Zaid Y., Guessous F., Puhm F. (2021). Platelet reactivity to thrombin differs between patients with COVID-19 and those with ARDS unrelated to COVID-19. Blood Adv.

[83] Cognasse F., Duchez A.C., Audoux E. (2022). Platelets as key factors in inflammation: Focus on CD40L/CD40. Front Immunol.

[84] Parry G.C.N., Martin T., Felts K.A., Cobb R.R. (1998). IL-1β–Induced monocyte chemoattractant protein-1 gene expression in endothelial cells is Blocked by proteasome inhibitors. Arterioscler Thromb Vasc Biol.

[85] Dole V.S., Bergmeier W., Patten I.S., Hirahashi J., Mayadas T.N., Wagner D.D. (2007). PSGL-1 regulates platelet P-selectin-mediated endothelial activation and shedding of P-selectin from activated platelets. Thromb Haemost.

[86] Ridker P.M., Buring J.E., Rifai N. (2001). Soluble P-selectin and the risk of future cardiovascular events. Circulation.

[87] Leucker T.M., Osburn W.O., Reventun P. (2021). Effect of crizanlizumab, a P-selectin inhibitor, in COVID-19: a placebo-controlled, randomized trial. JACC Basic Transl Sci.

[88] von Hundelshausen P., Koenen R.R., Sack M. (2005). Heterophilic interactions of platelet factor 4 and RANTES promote monocyte arrest on endothelium. Blood.

[89] Duerschmied D., Suidan G.L., Demers M. (2013). Platelet serotonin promotes the recruitment of neutrophils to sites of acute inflammation in mice. Blood.

[90] Corrales-Medina V.F., Madjid M., Musher D.M. (2010). Role of acute infection in triggering acute coronary syndromes. Lancet Infect Dis.

[91] Mallat Z., Corbaz A., Scoazec A. (2001). Interleukin-18/interleukin-18 binding protein signaling modulates atherosclerotic lesion development and stability. Circ Res.

[92] Hauer A.D., Uyttenhove C., de Vos P. (2005). Blockade of interleukin-12 function by protein vaccination attenuates atherosclerosis. Circulation.

[93] Frostegård J., Ulfgren A.K., Nyberg P. (1999). Cytokine expression in advanced human atherosclerotic plaques: dominance of proinflammatory (Th1) and macrophage-stimulating cytokines. Atherosclerosis.

[94] Kuan R., Agrawal D.K., Thankam F.G. (2021). Treg cells in atherosclerosis. Mol Biol Rep.

[95] Bayati A., Kumar R., Francis V., McPherson P.S. (2021). SARS-CoV-2 infects cells after viral entry via clathrin-mediated endocytosis. J Biol Chem.

[96] Kanneganti T.D. (2020). Intracellular innate immune receptors: Life inside the cell. Immunol Rev.

[97] Totura A.L., Whitmore A., Agnihothram S. (2015). Toll-like receptor 3 signaling via TRIF contributes to a protective innate immune response to severe acute respiratory syndrome coronavirus infection. mBio.

[98] Zheng M., Karki R., Williams E.P. (2021). TLR2 senses the SARS-CoV-2 envelope protein to produce inflammatory cytokines. Nat Immunol.

[99] Choudhury A., Mukherjee S. (2020). In silico studies on the comparative characterization of the interactions of SARS-CoV-2 spike glycoprotein with ACE-2 receptor homologs and human TLRs. J Med Virol.

[100] Hurst J., Prinz N., Lorenz M. (2009). TLR7 and TLR8 ligands and antiphospholipid antibodies show synergistic effects on the induction of IL-1beta and caspase-1 in monocytes and dendritic cells. Immunobiology.

[101] Yin X., Riva L., Pu Y. (2021). MDA5 Governs the innate immune response to SARS-CoV-2 in lung epithelial cells. Cell Rep.

[102] Grebe A., Hoss F., Latz E. (2018). NLRP3 inflammasome and the IL-1 pathway in atherosclerosis. Circ Res.

[103] Jiang C., Xie S., Yang G., Wang N. (2021). Spotlight on NLRP3 inflammasome: role in pathogenesis and therapies of atherosclerosis. J Inflamm Res.

[104] Vora S.M., Lieberman J., Wu H. (2021). Inflammasome activation at the crux of severe COVID-19. Nat Rev Immunol.

[105] Tardif J.C., Bouabdallaoui N., L’Allier P.L. (2021). Colchicine for community-treated patients with COVID-19 (COLCORONA): a phase 3, randomised, double-blinded, adaptive, placebo-controlled, multicentre trial. Lancet Respir Med.

[106] Lucas C., Wong P., Klein J. (2020). Longitudinal analyses reveal immunological misfiring in severe COVID-19. Nature.

[107] Langer H.F., Chavakis T. (2009). Leukocyte – endothelial interactions in inflammation. J Cell Mol Med.

[108] Henderson L.A., Canna S.W., Schulert G.S. (2020). On the Alert for cytokine storm: Immunopathology in COVID-19. Arthritis Rheumatol.

[109] Croca S., Rahman A. (2017). Atherosclerosis in systemic lupus erythematosus. Best Pract Res Clin Rheumatol.

[110] Ridker P.M., Everett B.M., Thuren T. (2017). Antiinflammatory therapy with Canakinumab for atherosclerotic disease. N Engl J Med.

[111] Morton A.C., Rothman A.M.K., Greenwood J.P. (2015). The effect of interleukin-1 receptor antagonist therapy on markers of inflammation in non-ST elevation acute coronary syndromes: the MRC-ILA Heart Study. Eur Heart J.

[112] Kyriazopoulou E., Poulakou G., Milionis H. (2021). Early treatment of COVID-19 with anakinra guided by soluble urokinase plasminogen receptor plasma levels: a double-blind, randomized controlled phase 3 trial. Nat Med.

[113] Potere N., Del Buono M.G., Caricchio R. (2022). Interleukin-1 and the NLRP3 inflammasome in COVID-19: Pathogenetic and therapeutic implications. EBioMedicine.

[114] Kleveland O., Kunszt G., Bratlie M. (2016). Effect of a single dose of the interleukin-6 receptor antagonist tocilizumab on inflammation and troponin T release in patients with non-ST-elevation myocardial infarction: a double-blind, randomized, placebo-controlled phase 2 trial. Eur Heart J.

[115] Broch K., Anstensrud A.K., Woxholt S. (2021). Randomized trial of interleukin-6 receptor inhibition in patients with acute ST-segment elevation myocardial infarction. J Am Coll Cardiol.

[116] Anon (2021). Interleukin-6 receptor antagonists in critically Ill patients with covid-19. N Engl J Med.

[117] Abani O., Abbas A., Abbas F. (2021). Tocilizumab in patients admitted to hospital with COVID-19 (RECOVERY): a randomised, controlled, open-label, platform trial. Lancet.

[118] Opstal T.S.J., Hoogeveen R.M., Fiolet A.T.L. (2020). Colchicine attenuates inflammation beyond the inflammasome in chronic coronary artery disease. Circulation.

[119] Fiolet A.T.L., Opstal T.S.J., Mosterd A. (2021). Efficacy and safety of low-dose colchicine in patients with coronary disease: a systematic review and meta-analysis of randomized trials. Eur Heart J.

[120] Murakami N., Hayden R., Hills T. (2023). Therapeutic advances in COVID-19. Nat Rev Nephrol.

[121] Attiq A., Afzal S., Ahmad W., Kandeel M. (2024). Hegemony of inflammation in atherosclerosis and coronary artery disease. Eur J Pharmacol.

[122] Anon (6 October 2021). A clinical case definition of post COVID-19 condition by a Delphi consensus. https://www.who.int/publications/i/item/WHO-2019-nCoV-Post_COVID-19_condition-Clinical_case_definition-2021.1.

[123] Nalbandian A., Sehgal K., Gupta A. (2021). Post-acute COVID-19 syndrome. Nat Med.

[124] Thaweethai T., Jolley S.E., Karlson E.W. (2023). Development of a definition of Postacute sequelae of SARS-CoV-2 infection. JAMA.

[125] Fischer A., Zhang L., Elbéji A. (2022). Long COVID Symptomatology after 12 Months and its impact on quality of Life According to initial coronavirus disease 2019 disease severity. Open Forum Infect Dis.

[126] Satterfield B.A., Bhatt D.L., Gersh B.J. (2021). Cardiac involvement in the long-term implications of COVID-19. Nat Rev Cardiol.

[127] Al-Aly Z., Xie Y., Bowe B. (2021). High-dimensional characterization of post-acute sequelae of COVID-19. Nature.

[128] Wang W., Wang S.-I., Wei J.C.-C. (2022). Response to: ‘Concerns about ‘Long-term cardiovascular outcomes in COVID-19 survivors among non-vaccinated population: A retrospective cohort study from the TriNetX US collaborative networks’ by Renin Chang et al. EClinicalMedicine.

[129] Xie Y., Xu E., Bowe B., Al-Aly Z. (2022). Long-term cardiovascular outcomes of COVID-19. Nat Med.

[130] Buckley B.J.R., Harrison S.L., Fazio-Eynullayeva E. (2021). Prevalence and clinical outcomes of myocarditis and pericarditis in 718,365 COVID-19 patients. Eur J Clin Invest.

[131] Katsoularis I., Fonseca-Rodríguez O., Farrington P. (2021). Risk of acute myocardial infarction and ischaemic stroke following COVID-19 in Sweden: a self-controlled case series and matched cohort study. Lancet.

[132] Kim Y.E., Huh K., Park Y.J., Peck K.R., Jung J. (2022). Association Between Vaccination and Acute Myocardial Infarction and Ischemic Stroke After COVID-19 Infection. JAMA.

[133] Knight R., Walker V., Ip S. (2022). Association of COVID-19 With Major Arterial and Venous Thrombotic Diseases: A Population-Wide Cohort Study of 48 Million Adults in England and Wales. Circulation.

[134] Wiemken T.L., McGrath L.J., Andersen K.M. (2022). Coronavirus Disease 2019 Severity and Risk of Subsequent Cardiovascular Events. Clinical Infectious Diseases.

[135] Wan E.Y.F., Mathur S., Zhang R. (2023). Association of COVID-19 with short- and long-term risk of cardiovascular disease and mortality: a prospective cohort in UK Biobank. Cardiovasc Res.

[136] Raisi-Estabragh Z., Cooper J., Salih A. (2023). Cardiovascular disease and mortality sequelae of COVID-19 in the UK Biobank. Heart.

[137] DeVries A., Shambhu S., Sloop S., Overhage J.M. (2023). One-Year Adverse Outcomes Among US Adults With Post–COVID-19 Condition vs Those Without COVID-19 in a Large Commercial Insurance Database. JAMA Health Forum.

[138] Koyama A.K., Imperatore G., Rolka D.B. (2023). Risk of Cardiovascular Disease After COVID-19 Diagnosis Among Adults With and Without Diabetes. J Am Heart Assoc.

[139] Kim Y.E., Huh K., Park Y.J., Peck K.R., Jung J. (2022). Association between vaccination and acute myocardial infarction and ischemic stroke after COVID-19 infection. JAMA.

[140] Florescu S., Stanciu D., Zaharia M. (2023). Long-term (180-Day) outcomes in critically Ill patients with COVID-19 in the REMAP-CAP randomized clinical trial. JAMA.

[141] Chertow D., Stein S., Ramelli S. (Published online December 20, 2021). SARS-CoV-2 infection and persistence throughout the human body and brain. Res Square.

[142] Natarajan A., Zlitni S., Brooks E.F. (2022). Gastrointestinal symptoms and fecal shedding of SARS-CoV-2 RNA suggest prolonged gastrointestinal infection. Med.

[143] Newell K.L., Waickman A.T. (2022). Inflammation, immunity, and antigen persistence in post-acute sequelae of SARS-CoV-2 infection. Curr Opin Immunol.

[144] Peluso M.J., Deeks S.G. (2022). Early clues regarding the pathogenesis of long-COVID. Trends Immunol.

[145] Braun J., Loyal L., Frentsch M. (2020). SARS-CoV-2-reactive T cells in healthy donors and patients with COVID-19. Nature.

[146] Gaebler C., Wang Z., Lorenzi J.C.C. (2021). Evolution of antibody immunity to SARS-CoV-2. Nature.

[147] Taeschler P., Adamo S., Deng Y. (2022). T-cell recovery and evidence of persistent immune activation 12 months after severe COVID-19. Allergy.

[148] Cirelli K.M., Crotty S. (2017). Germinal center enhancement by extended antigen availability. Curr Opin Immunol.

[149] Files J.K., Sarkar S., Fram T.R. (2021). Duration of post–COVID-19 symptoms is associated with sustained SARS-CoV-2–specific immune responses. JCI Insight.

[150] Cervia C., Zurbuchen Y., Taeschler P. (2022). Immunoglobulin signature predicts risk of post-acute COVID-19 syndrome. Nat Commun.

[151] Antonelli M., Penfold R.S., Merino J. (2022). Risk factors and disease profile of post-vaccination SARS-CoV-2 infection in UK users of the COVID Symptom Study app: a prospective, community-based, nested, case-control study. Lancet Infect Dis.

[152] Arnold D.T., Milne A., Samms E., Stadon L., Maskell N.A., Hamilton F.W. (2021). Symptoms after COVID-19 vaccination in patients with persistent symptoms after acute infection: a case series. Ann Intern Med.

[153] André S., Picard M., Cezar R. (2022). T cell apoptosis characterizes severe Covid-19 disease. Cell Death Differ.

[154] Acosta-Ampudia Y., Monsalve D.M., Rojas M. (2022). Persistent autoimmune activation and proinflammatory state in post-coronavirus disease 2019 syndrome. J Infect Dis.

[155] Orologas-Stavrou N., Politou M., Rousakis P. (2020). Peripheral blood immune profiling of convalescent plasma donors reveals alterations in specific immune Subpopulations even at 2 Months post SARS-CoV-2 infection. Viruses.

[156] Choy J.C., McDonald P.C., Suarez A.C. (2003). Granzyme B in atherosclerosis and transplant vascular disease: association with cell death and atherosclerotic disease severity. Mod Pathol.

[157] Richter A.G., Shields A.M., Karim A. (2021). Establishing the prevalence of common tissue-specific autoantibodies following severe acute respiratory syndrome coronavirus 2 infection. Clin Exp Immunol.

[158] Peluso M.J., Mitchell A., Wang C.Y. (2022). Low prevalence of interferon α autoantibodies in People experiencing symptoms of post–coronavirus disease 2019 (COVID-19) Conditions, or long COVID. J Infect Dis.

[159] Bertin D., Kaphan E., Weber S. (2021). Persistent IgG anti-cardiolipin autoantibodies are associated with post-COVID syndrome. Int J Infect Dis.

[160] Son K., Jamil R., Chowdhury A. (2022). Circulating anti-nuclear autoantibodies in COVID-19 survivors predict long-COVID symptoms. Eur Respir J.

[161] Altmann D.M., Reynolds C.J., Joy G. (2023). Persistent symptoms after COVID-19 are not associated with differential SARS-CoV-2 antibody or T cell immunity. Nat Commun.

[162] Fogarty H., Townsend L., Morrin H. (2021). Persistent endotheliopathy in the pathogenesis of long COVID syndrome. J Thromb Haemost.

[163] Evans P.C., Ed Rainger G., Mason J.C. (2020). Endothelial dysfunction in COVID-19: a position paper of the ESC Working group for atherosclerosis and vascular biology, and the ESC Council of basic cardiovascular Science. Cardiovasc Res.

[164] Thijssen D.H.J., Bruno R.M., van Mil A.C.C.M. (2019). Expert consensus and evidence-based recommendations for the assessment of flow-mediated dilation in humans. Eur Heart J.

[165] Xu Y., Arora R.C., Hiebert B.M. (2014). Non-invasive endothelial function testing and the risk of adverse outcomes: a systematic review and meta-analysis. Eur Heart J Cardiovasc Imaging.

[166] Ratchford S.M., Stickford J.L., Province V.M. (2021). Vascular alterations among young adults with SARS-CoV-2. Am J Physiol Heart Circ Physiol.

[167] Ambrosino P., Calcaterra I., Molino A. (2021). Persistent endothelial dysfunction in post-acute covid-19 syndrome: A case-control study. Biomedicines.

[168] Oikonomou E., Souvaliotis N., Lampsas S. (2022). Endothelial dysfunction in acute and long standing COVID−19: A prospective cohort study. Vascul Pharmacol.

[169] Riou M., Oulehri W., Momas C. (2021). Reduced Flow-Mediated Dilatation Is Not Related to COVID-19 Severity Three Months after Hospitalization for SARS-CoV-2 Infection. J Clin Med.

[170] Santoro L., Falsetti L., Zaccone V. (2022). Impaired Endothelial Function in Convalescent Phase of COVID-19: A 3 Month Follow Up Observational Prospective Study. J Clin Med.

[171] Nandadeva D., Young B.E., Stephens B.Y. (2021). Blunted peripheral but not cerebral vasodilator function in young otherwise healthy adults with persistent symptoms following COVID-19. Am J Physiol Heart Circ Physiol.

[172] Szeghy R.E., Province V.M., Stute N.L. (2022). Carotid stiffness, intima–media thickness and aortic augmentation index among adults with SARS-CoV-2. Exp Physiol.

[173] Zanoli L., Gaudio A., Mikhailidis D.P. (2022). Vascular Dysfunction of COVID-19 Is Partially Reverted in the Long-Term. Circ Res.

[174] Weber B., Parks S., Huck D.M. (2022). Prior SARS-CoV-2 Infection Is Associated With Coronary Vasomotor Dysfunction as Assessed by Coronary Flow Reserve From Cardiac Positron Emission Tomography. J Am Heart Assoc.

[175] Tong M., Yan X., Jiang Y. (2022). Endothelial Biomarkers in Patients Recovered from COVID-19 One Year after Hospital Discharge: A Cross-Sectional Study. Mediterr J Hematol Infect Dis.

[176] Charfeddine S., Ibn Hadj Amor H., Jdidi J. (2021). Long COVID 19 Syndrome: Is It Related to Microcirculation and Endothelial Dysfunction? Insights From TUN-EndCOV Study. Front Cardiovasc Med.

[177] Poyatos P., Luque N., Eizaguirre S. (2022). Post-COVID-19 patients show an increased endothelial progenitor cell production. Transl Res.

[178] Verma A., Ramayya T., Anand U. (2022). Post COVID-19 syndrome with impairment of flow-mediated epicardial vasodilation and flow reserve. Eur J Clin Invest.

[179] Vollbracht C., Kraft K. (2022). Oxidative stress and hyper-inflammation as major drivers of severe COVID-19 and long COVID: implications for the benefit of high-dose Intravenous Vitamin C. Front Pharmacol.

[180] Guervilly C., Burtey S., Sabatier F. (2020). The Journal of infectious diseases circulating endothelial cells as a marker of endothelial injury in severe COVID-19. J Infect Dis.

[181] Chioh F.W.J., Fong S.W., Young B.E. (2021). Convalescent covid-19 patients are susceptible to endothelial dysfunction due to persistent immune activation. Elife.

[182] Peluso M.J., Lu S., Tang A.F. (2021). Markers of immune activation and inflammation in individuals with Postacute sequelae of severe acute respiratory syndrome coronavirus 2 infection. J Infect Dis.

[183] Phetsouphanh C., Darley D.R., Wilson D.B. (2022). Immunological dysfunction persists for 8 months following initial mild-to-moderate SARS-CoV-2 infection. Nat Immunol.

[184] Sollini M., Ciccarelli M., Cecconi M. (2021). Vasculitis changes in COVID-19 survivors with persistent symptoms: an [18F]FDG-PET/CT study. Eur J Nucl Med Mol Imaging.

[185] Mátyás B.B., Benedek I., Blîndu E. (2023). Elevated FAI index of Pericoronary inflammation on coronary CT Identifies increased risk of coronary plaque Vulnerability after COVID-19 infection. Int J Mol Sci.

[186] Karadavut S., Altintop I. (2022). Long-term cardiovascular adverse events in very elderly COVID-19 patients. Arch Gerontol Geriatr.

[187] Su Y., Yuan D., Chen D.G. (2022). Multiple early factors anticipate post-acute COVID-19 sequelae. Cell.

[188] Virani S.S., Newby L.K., Arnold S.V. (2023). 2023 AHA/ACC/ACCP/ASPC/NLA/PCNA guideline for the management of patients with chronic coronary disease: a report of the American heart association/American College of Cardiology Joint Committee on clinical Practice guidelines. Circulation.

[189] Jovanoski N., Chen X., Becker U. (2021). Severity of COVID-19 and adverse long-term outcomes: a retrospective cohort study based on a US electronic health record database. BMJ Open.

[190] Knuuti J., Ballo H., Juarez-Orozco L.E. (2018). The performance of non-invasive tests to rule-in and rule-out significant coronary artery stenosis in patients with stable angina: a meta-analysis focused on post-test disease probability. Eur Heart J.

